# Supplementary material for: Internalized components of membrane attack complexes disrupt proteostasis and acquire alarmin-like properties
Source: Nat Commun. 2026 Jul 17;17:8770. doi: 10.1038/s41467-026-75696-0 (PMC13494016; doi:10.1038/s41467-026-75696-0)
Supplement: Supplementary file 1 [file 41467_2026_75696_MOESM1_ESM.pdf]

SUPPLEMENTARY INFORMATION:

Internalized Components of Membrane Attack Complexes Disrupt Proteostasis and Acquire Alarmin-Like Properties

Guiyu Song,[1,2,3,¶,*] Zihan Ma,[1,2,¶] Matthew Fan,[2,¶] Liying He,[1,2] Yulong Lan,[4] Weihao Li,[5] Zhengtao Jiang,[1,2] Quan Jiang,[1,2] Dylan P. Noone,[6] Andrea Nans,[7] Kamal L. Nahas,[8] Mahsa Nouri Barkestani,[1,2] Shaoxun Wang,[1,9] Qianxun Wang,[1,2] Pengwei Ren,[9] Jolin Cheng,[2] Yinuo Zang,[2] Haitian Zhou,[2] Justin Johnson,[10] Clancy Mullan,[9,10] Xiangyu Gong,[11,12] Doryen Bubeck,[6,13] Gilbert Moeckel,[14] Michael Mak,[11,12] George Tellides,[9] Dan Jane-wit[1,2,*]

[1]Department of Cardiology, West Haven VA Medical Center, West Haven, CT, USA; [2]Section of Cardiovascular Medicine, Yale University School of Medicine, New Haven, CT, USA; [3]Department of Obstetrics and Gynecology, Shengjing Hospital of China Medical University, Shenyang, China; [4]Department of Neurosurgery, Second Affiliated Hospital, School of Medicine, Zhejiang University, Hangzhou, Zhejiang, China; [5]Department of Vascular Surgery, Peking University People's Hospital, Beijing, China; [6]Department of Life Sciences, Imperial College, London, UK; [7]Structural Biology Science Technology Platform, The Francis Crick Institute, London, NW1 9 1AT, UK; [8]Beamline B24, Diamond Light Source, Harwell Science and Innovation Campus, Didcot, United Kingdom; [9]Dept of Surgery; Yale University School of Medicine, New Haven, CT, USA; [10]Department of Immunobiology, Yale University School of Medicine, New Haven, CT, USA; [11]Dept of Biomedical Engineering, Yale University School of Medicine, New Haven, CT, USA; [12]Dept of Pharmacological Sciences, Stony Brook University, Stony Brook, NY, USA; [13]The Francis Crick Institute, London, NW1 1AT, UK. [14]Dept of Pathology, Yale University School of Medicine, New Haven, CT, USA; [¶]These authors contributed equally to the work. *Corresponding authors (songgy77@hotmail.com, dan.jane-wit@yale.edu)

SUPPLEMENTARY FIGURES

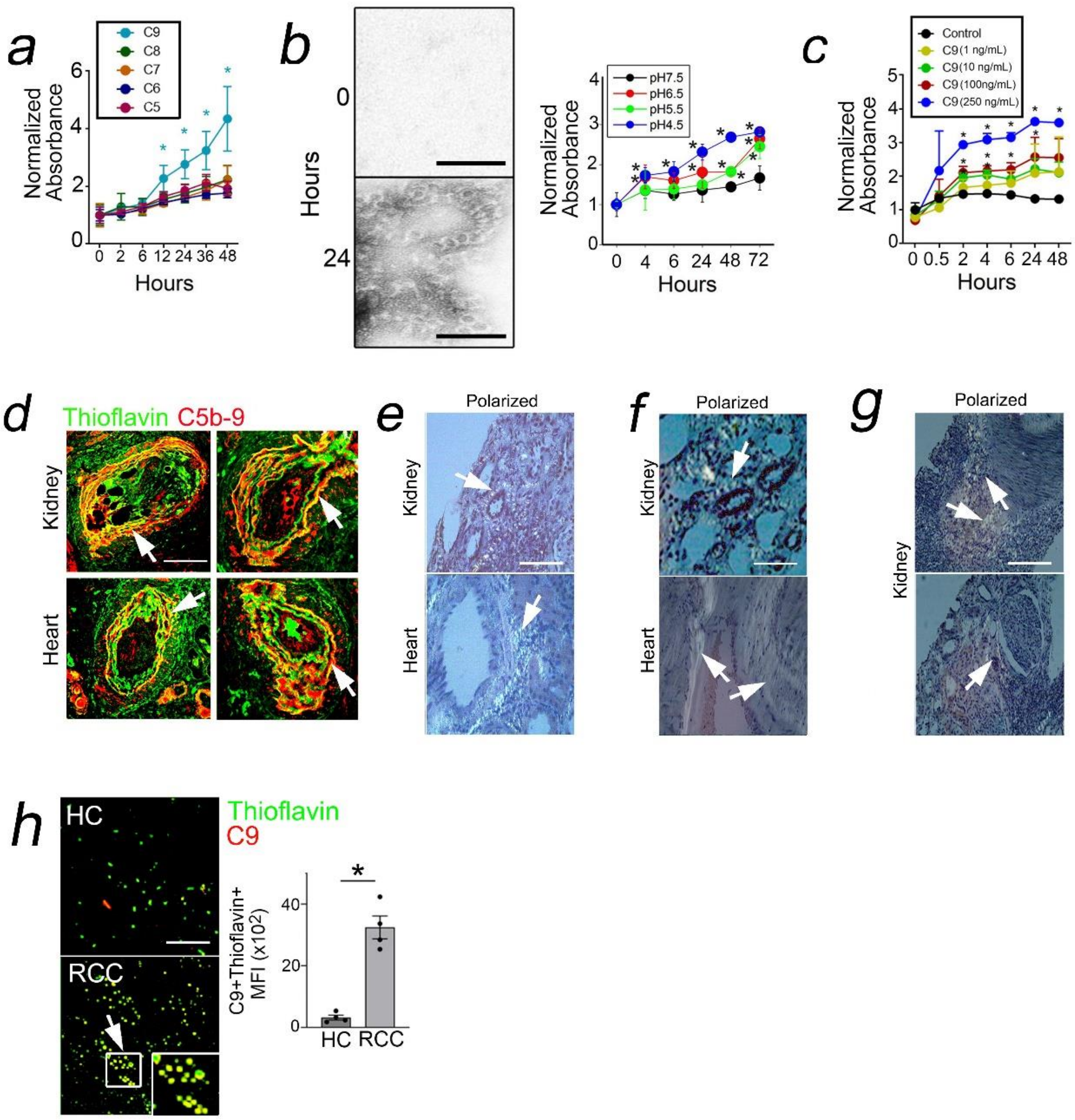

Supplementary Figure 1. Human complement proteins at 0.125 μg/mL were incubated at times indicated at pH 4.5 at 37°C (*a*). Human C9 protein (0.125 μg/mL) was resuspended in buffers of varying pH, and thioflavin fluorescence was assessed over time at 37°C (*b*). Human C9 protein at concentrations indicated were incubated at 37°C and thioflavin fluorescence was assessed over time (*c*). Human kidney and heart biopsies with CABMR were analyzed for thioflavin staining by I.F. (*d*, n=12). Kidney and heart biopsies with $C4d^-$ ABMR showing adventitial Congo red staining (*e,f*). Kidney biopsies with TCMR showing interstitial Congo Red staining (*g*). Kidney tissues from healthy controls (HC) and renal cell carcinoma (RCC) were analyzed by I.F. (*h*, n=4 per group). Experiments repeated ≥3 times using different HUVEC donors. Scale bars = 20nm (*b*), 200μm (*d*), and 100μm (*e-h*). Data points indicate technical replicates (*a-c*) or individual patient samples (*h*). * $p<0.05$ using two-way ANOVA (*a-c*) with Tukey's post-hoc correction or paired Student's *t*-test (*h*).

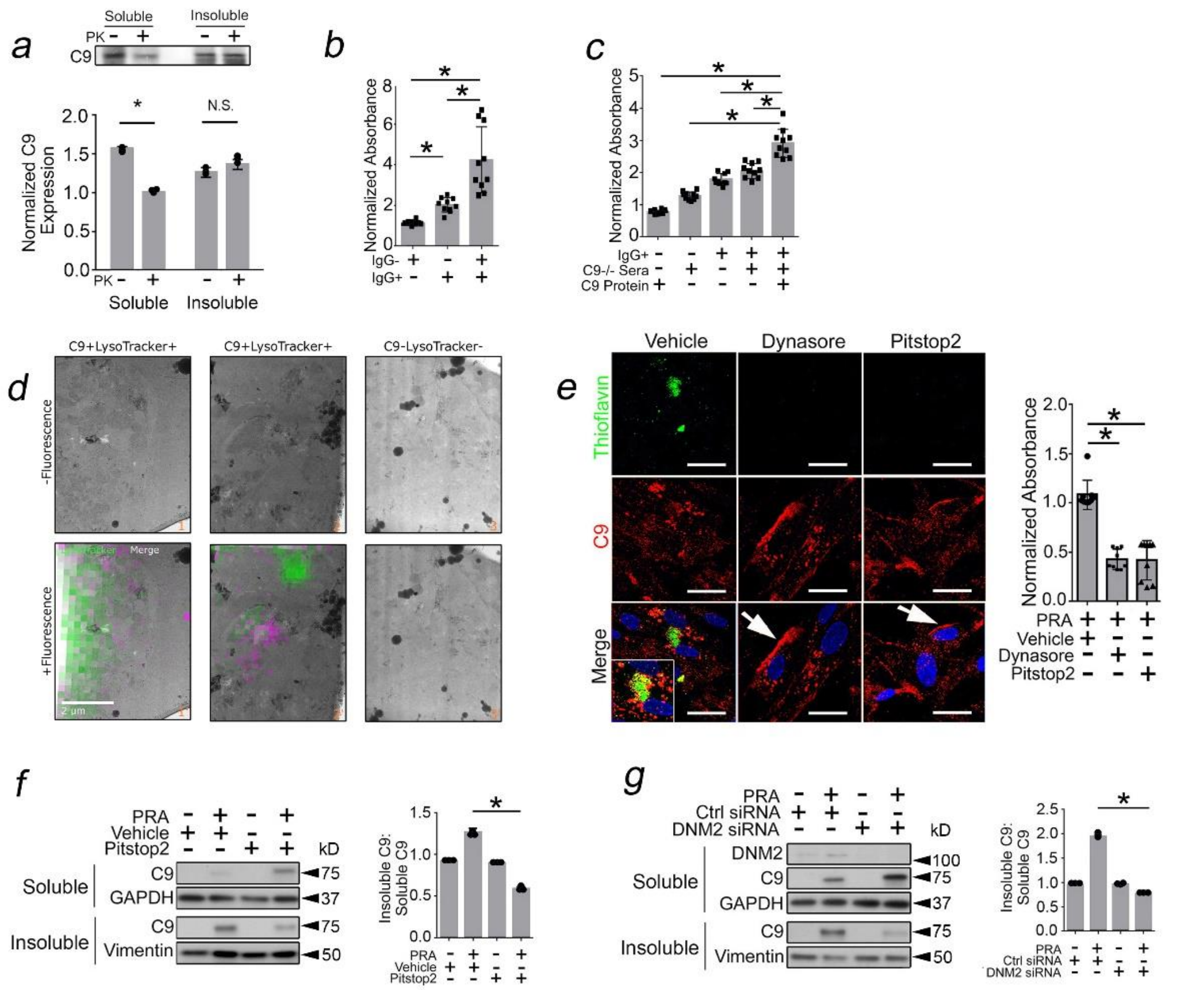

Supplementary Figure 2. SDS soluble and insoluble fractions of human C9 (0.125 μg/mL) were incubated at room temperature with proteinase K (12.5 μg/ml μg/mL) for 30 min prior to Western blot (*a*). C9 was normalized to GAPDH (lanes 1,2) or vimentin (lanes 3 and 4). PRA sera were fractionated and added to HUVECs for 2 hrs prior to assessing thioflavin fluorescence (*b*). IgG^+ fractions of PRA sera were added to HUVECs with $C9^{-/-}$ sera and C9 protein (5 μg/mL) for 2 hrs prior to assessing thioflavin fluorescence (*c*). Additional 2D images of milled lamellae used for cryoelectron tomography and for quantification, in addition to regions shown in Main Figure 2*h* (*d*). A negative-control field lacking C9 and LysoTracker staining is shown (*d*, right panel). The correlated fluorescence overlays show signal for both C9 (purple) and LysoTracker (green). Images 1 and 1′, 2 and 2′, and 3 and 3′ show the same fields of view without and with the fluorescence overlay, respectively. Fluorescence and electron microscopy modalities were aligned using three-point reference alignment in the MAPS software. HUVECs were pre-treated with vehicle, Dynasore (80 μg/mL), or Pitstop2 (30 μg/mL) for 30 min prior to addition of PRA for 4 hrs (*e*). Soluble and insoluble fractions of PRA-treated HUVECs exposed to vehicle or Pitstop2 were assessed for C9 (*f*). HUVECs transfected with control or DNM2 siRNA were treated with PRA, and soluble and insoluble lysates were assessed for C9 (*g*). Scale bars = 2μm (*d*) or 30μm (*e*). Experiments repeated ≥3 times using different HUVEC donors. Data points indicate technical replicates (*a-c,e-g*). * $p<0.05$ using one-way ANOVA (*e-g*) or two-way ANOVA (*b-c*) with Tukey's post-hoc correction or Student's *t*-test (*a*).

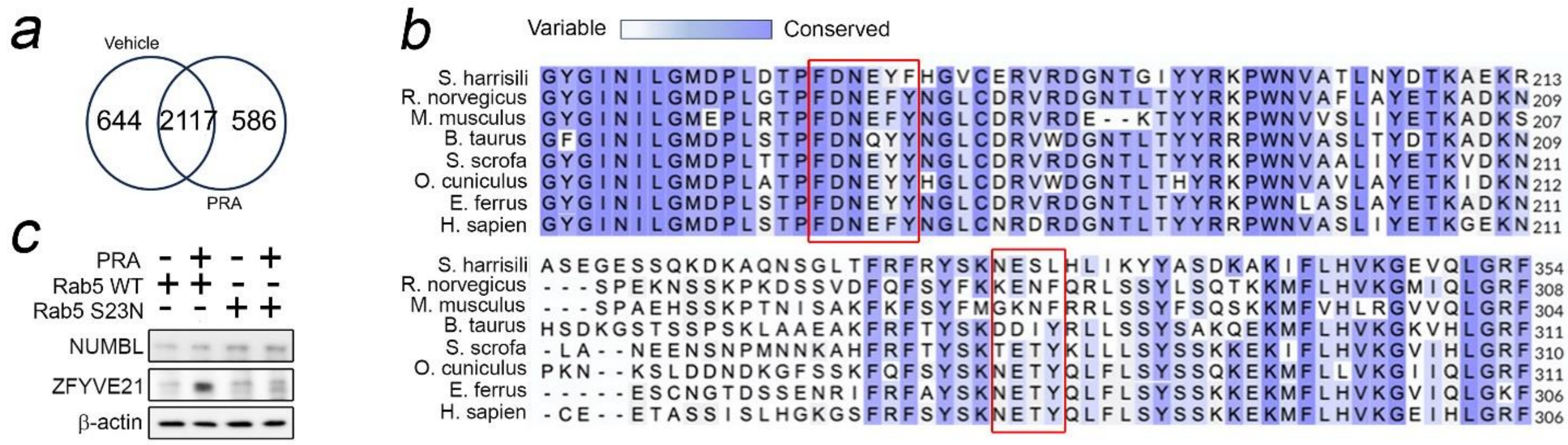

Supplementary Figure 3. ZFYVE21-GFP HUVECs were treated with PRA for 2 hours prior to GFP bead pulldowns and LC-MS/MS (*a,* n=3 runs). Sequence alignments predicted 2 NUMBL binding motifs on C9 (*b*). Western blots of HUVECs treated with PRA for 1 hour (*c*).

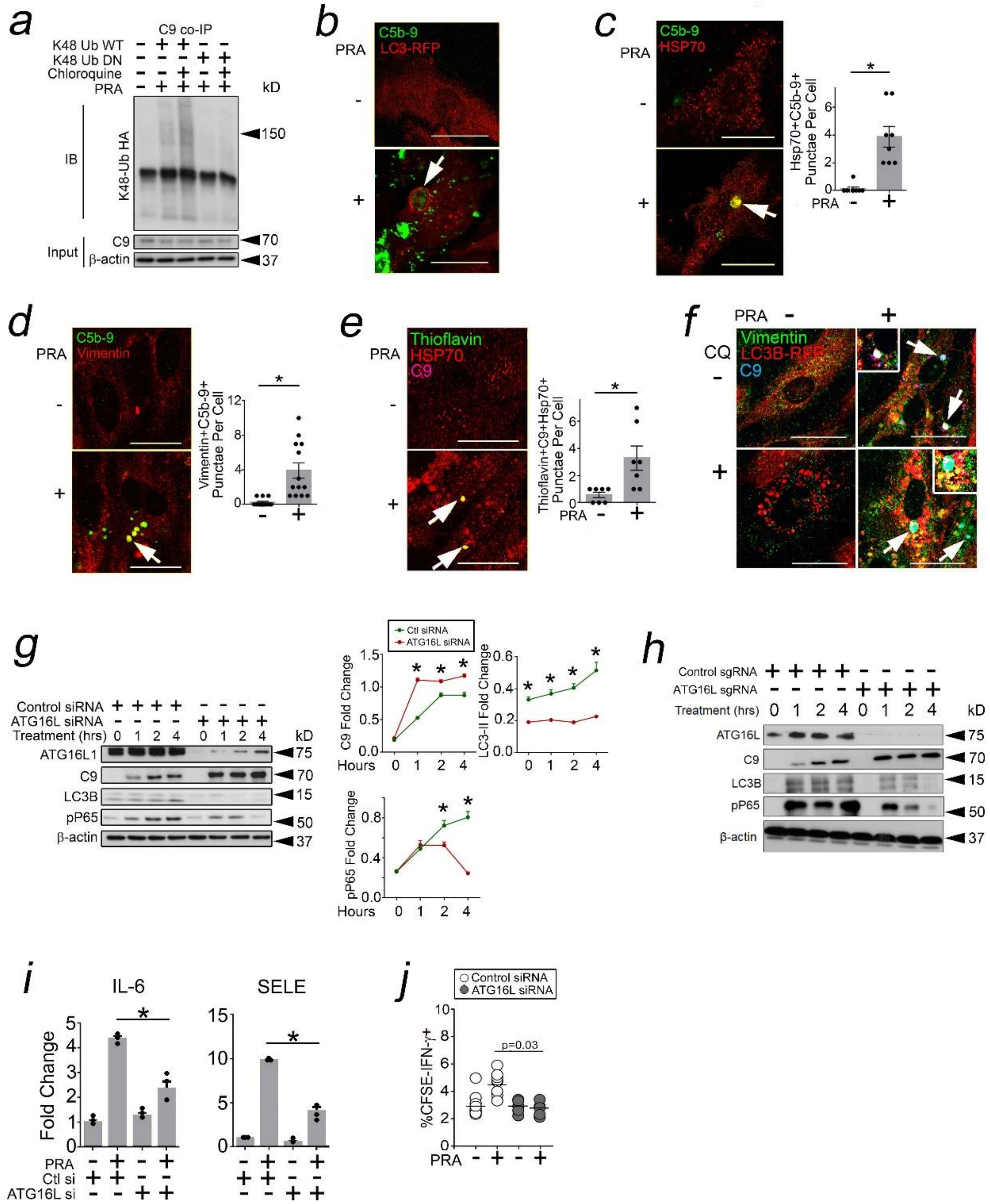

Supplementary Figure 4. K48 Ub-WT-HA was incubated with ZFYVE21, C9, and P62 proteins as indicated for 4 hours at 4°C prior to C9 pulldowns and Western blot analysis (*a*). HUVECs were treated with PRA for 2 hours prior to I.F. (*b-f*). HUVECs were transfected with ATG16L siRNA prior to pulse-chase studies with PRA (*g*). CRISPR/Cas9-mediated knockout of ATG16L was performed prior to pulse-chase studies with PRA (*h*). HUVECs were transfected with ATG16L siRNA prior to qRT-PCR (*i*), and EC:T cell cocultures (*j*) Data points indicate individual cells analyzed (*c-e*) or biological replicates (*i,j*). Scale bars = 30μm (*b-f*). * $p<0.05$ using one-way ANOVA (*i-j*) or two-way ANOVA (*g*) with Tukey's post-hoc correction or Student's *t*-test (*c-e*).

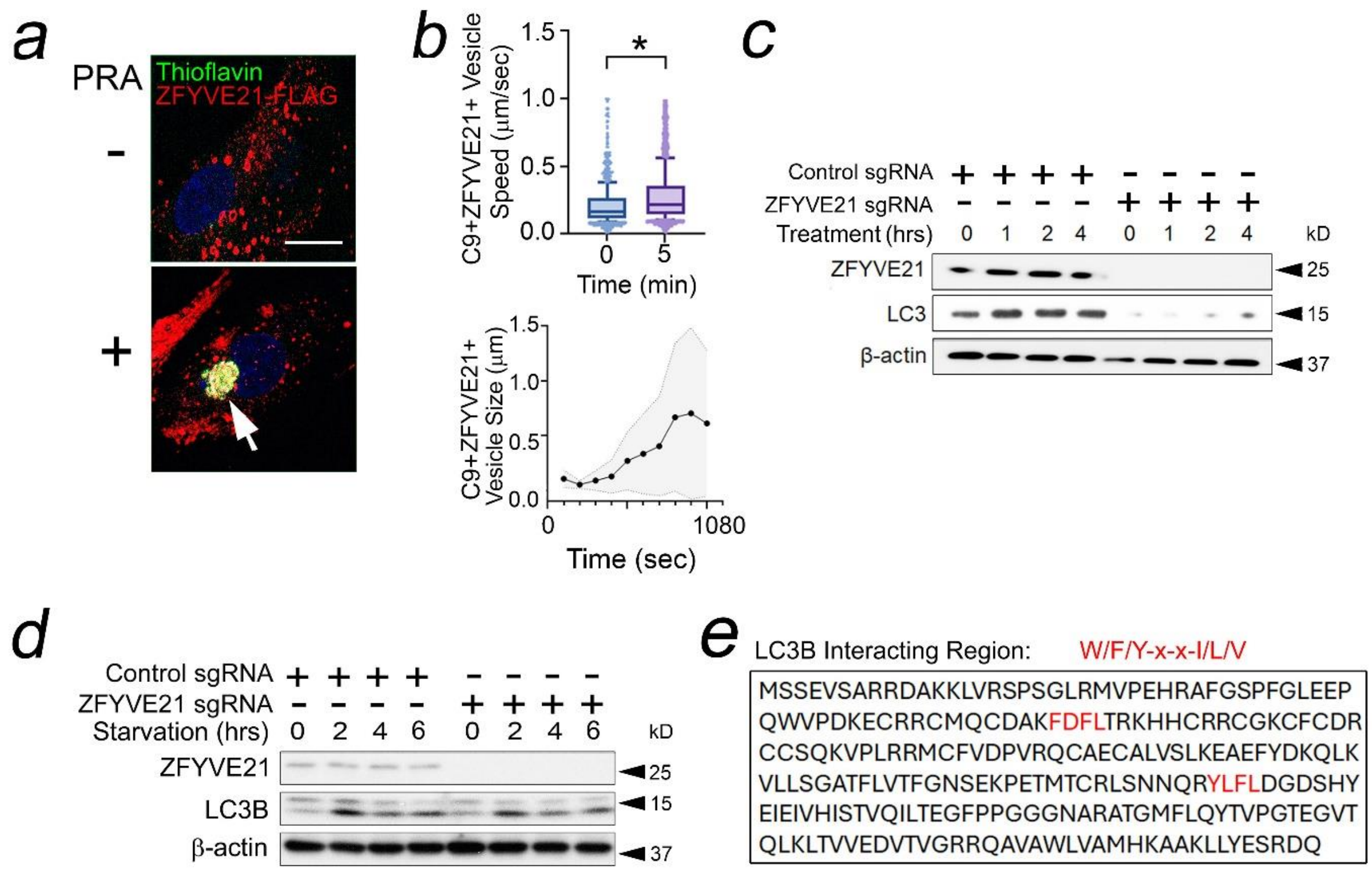

Supplementary Figure 5. HUVEC were stained as indicated after 2 hours of PRA treatment (*a*). Mean displacement (*b,* top) and mean size (bottom) for C9^{+}ZFYVE21^{+} vesicles were calculated in live cell imaging studies. HUVECs were treated with PRA (*c*) or serum starved (*d*) as indicated prior to use in pulse-chase studies. Human ZFYVE21 protein sequence contains two LC3B interacting regions (*e*). Scale bar = 30µm (*a*). Data points indicate individual cells analyzed (*b*) or biological replicates (*c,d*). * p<0.05 using two-way ANOVA (*d*) with Tukey's post-hoc correction or Student's *t*-test (*b*).

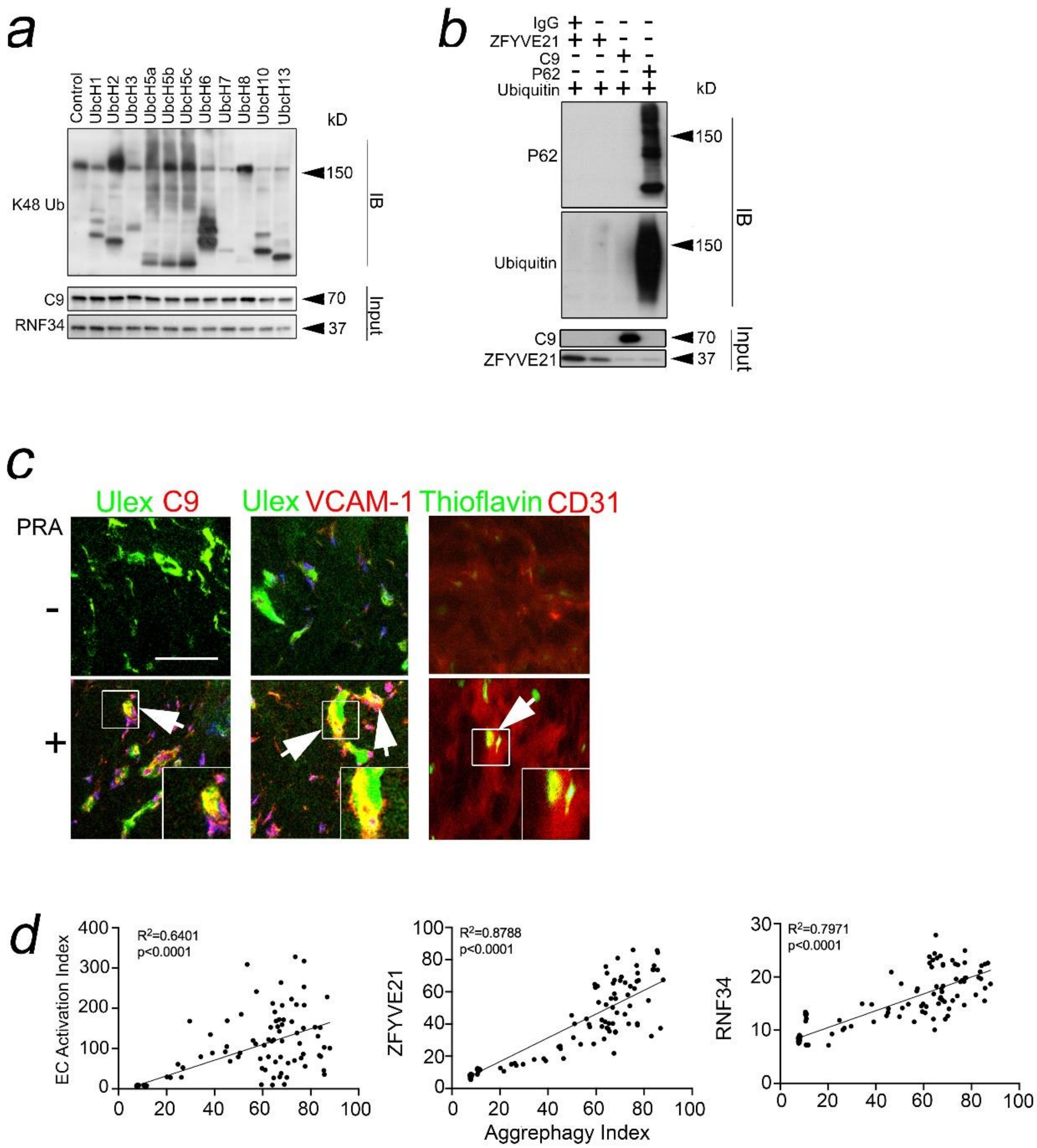

Supplementary Figure 6. Various E2 ubiquitin ligases were co-incubated with ubiquitin, E1 enzymes, RNF34 protein, and C9 protein and tested for *in vitro* ubiquitinylation of C9 for 4 hours at 37˚C (*a*). ZFYVE21, C9, or P62 proteins were incubated with Ub WT prior to Western blot analysis (*b*). HUVECs embedded in collagen-fibronectin gels and implanted in SCID/beige mice for 3 weeks prior to intravenous injection with 200µL neat PRA sera and gel harvest 24 hours later (*c,* n=3 per group). Correlation coefficients for genes indicated were calculated using public RNA-seq datasets (*d*). Scale bar = 20µm (*c*).

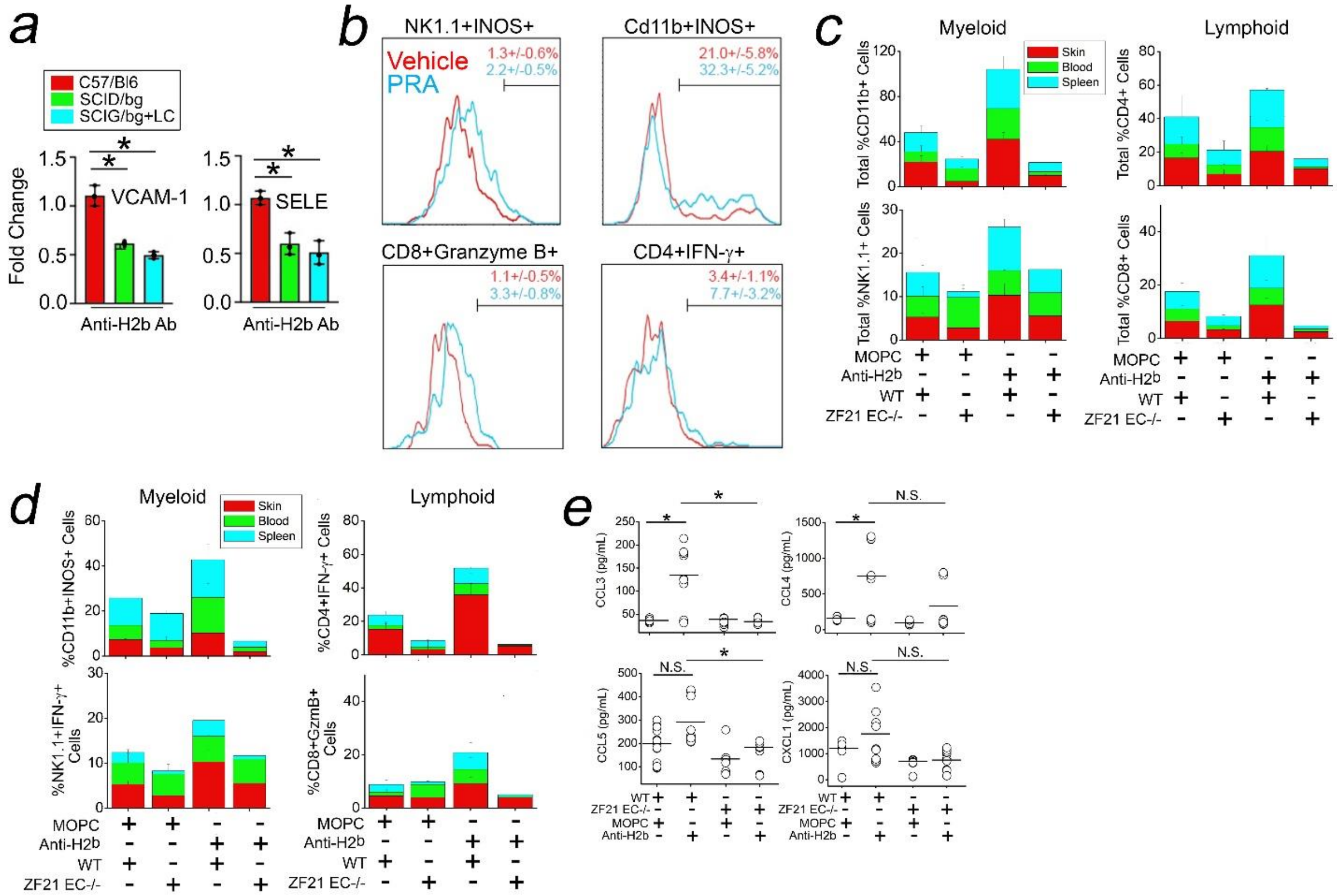

Supplementary Figure 7. qRT-PCR (*a*) and FACS analyses (*b*) were performed as indicated. Myeloid and lymphoid lineages expressed as a percent of total $CD45^{+}$ cells were analyzed in skin, blood, and splenic tissue compartments (*c,d*). Sera cytokine analysis (*e*). Data points indicate biological replicates (*a*, *e*). * p<0.05 using one-way ANOVA (*a*) or two-way ANOVA (*e*) with Tukey's post-hoc correction.

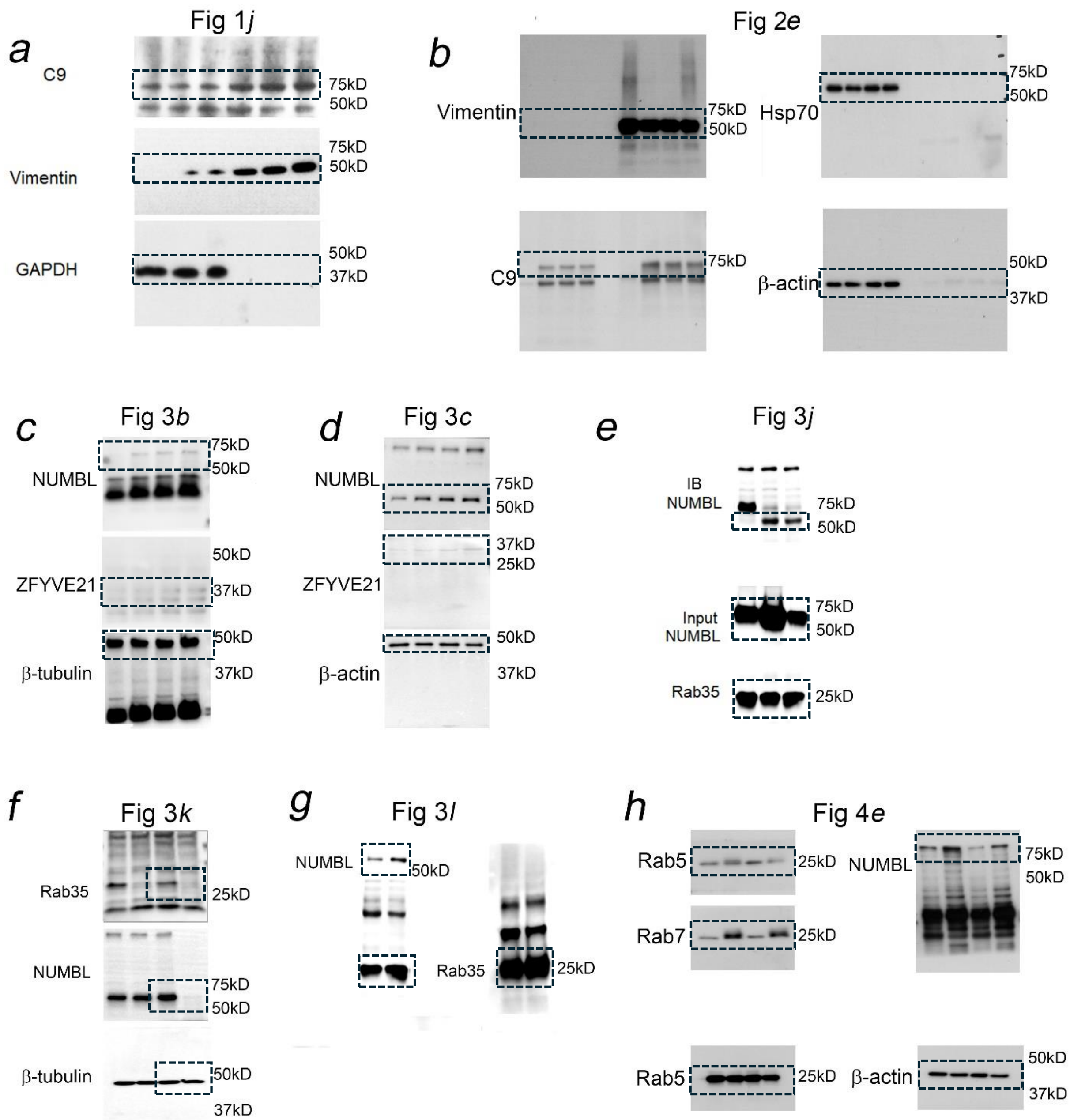

Supplementary Figure 8. Original uncropped films corresponding to Western blots in main Figures 1-4 in the manuscript.

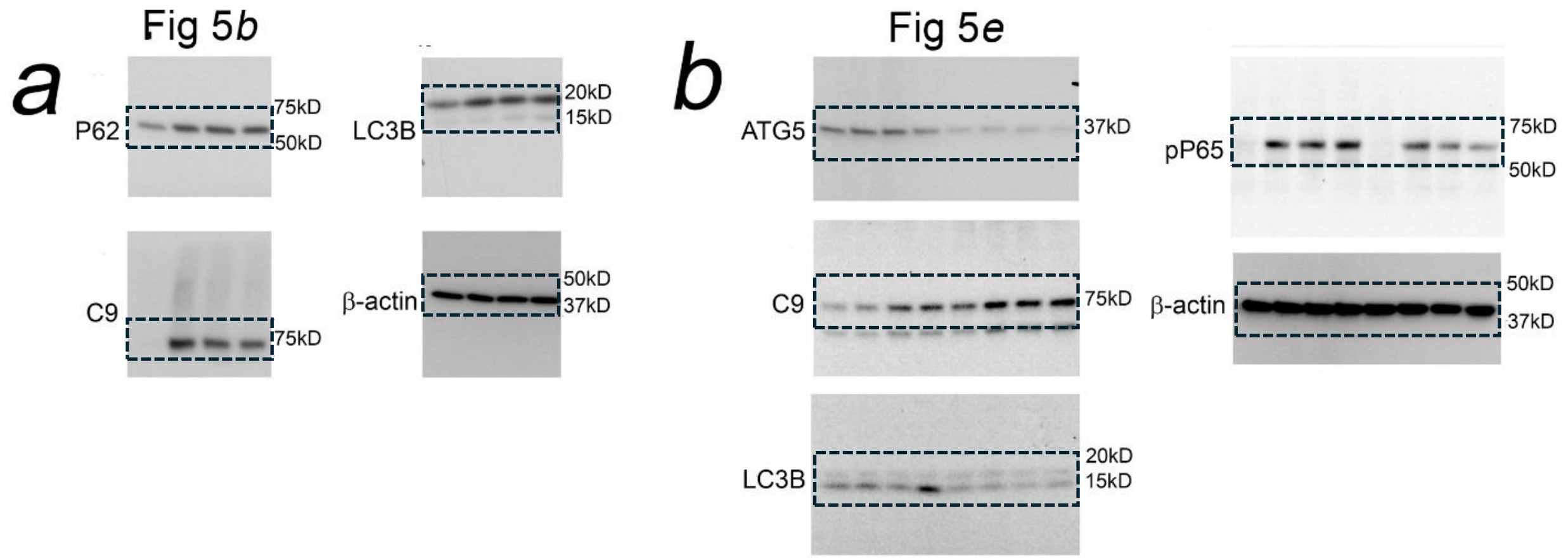

Supplementary Figure 9. Original uncropped films corresponding to Western blots in main Figure 5 in the manuscript.

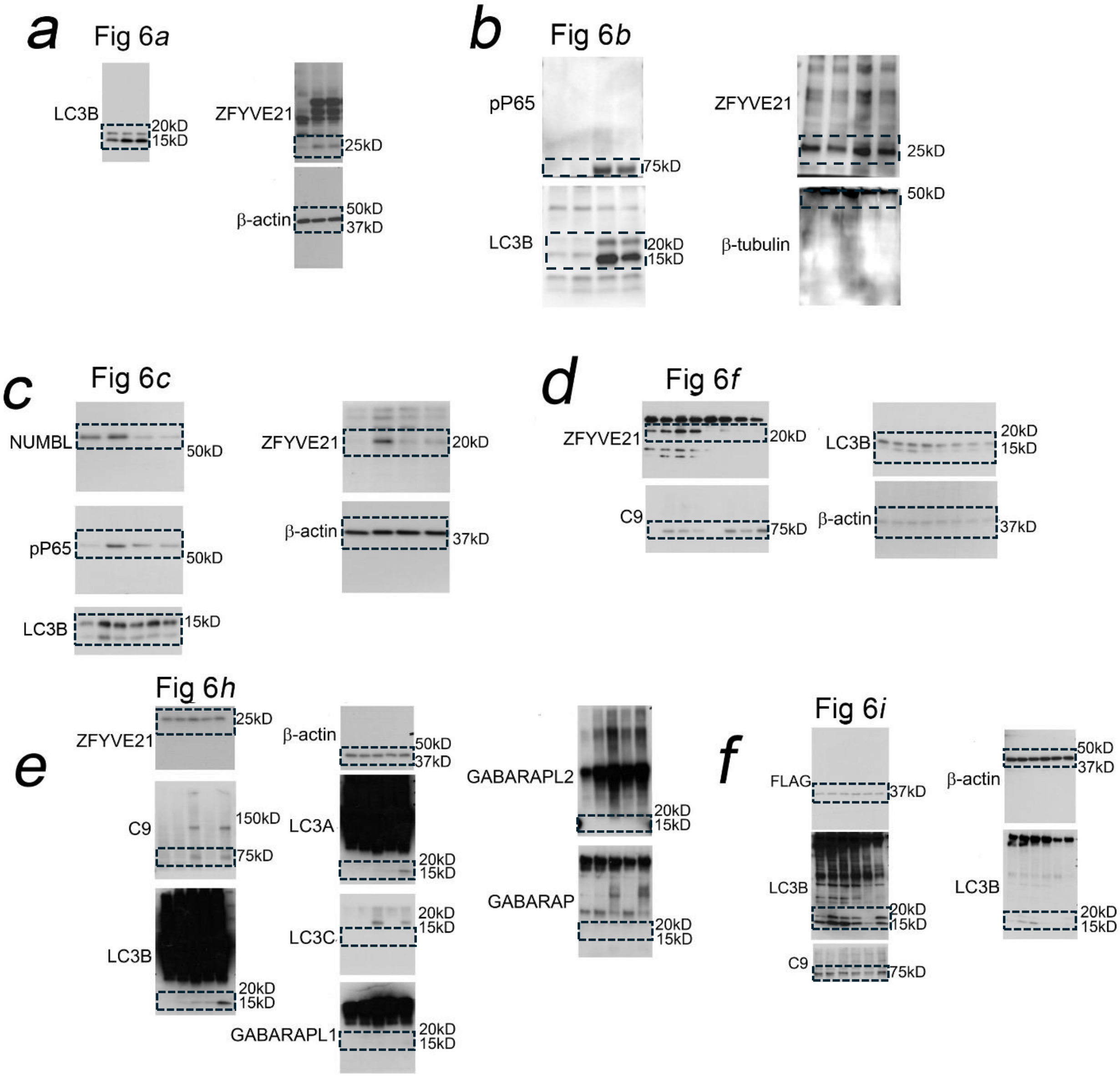

Supplementary Figure 10. Original uncropped films corresponding to Western blots in main Figure 6 in the manuscript.

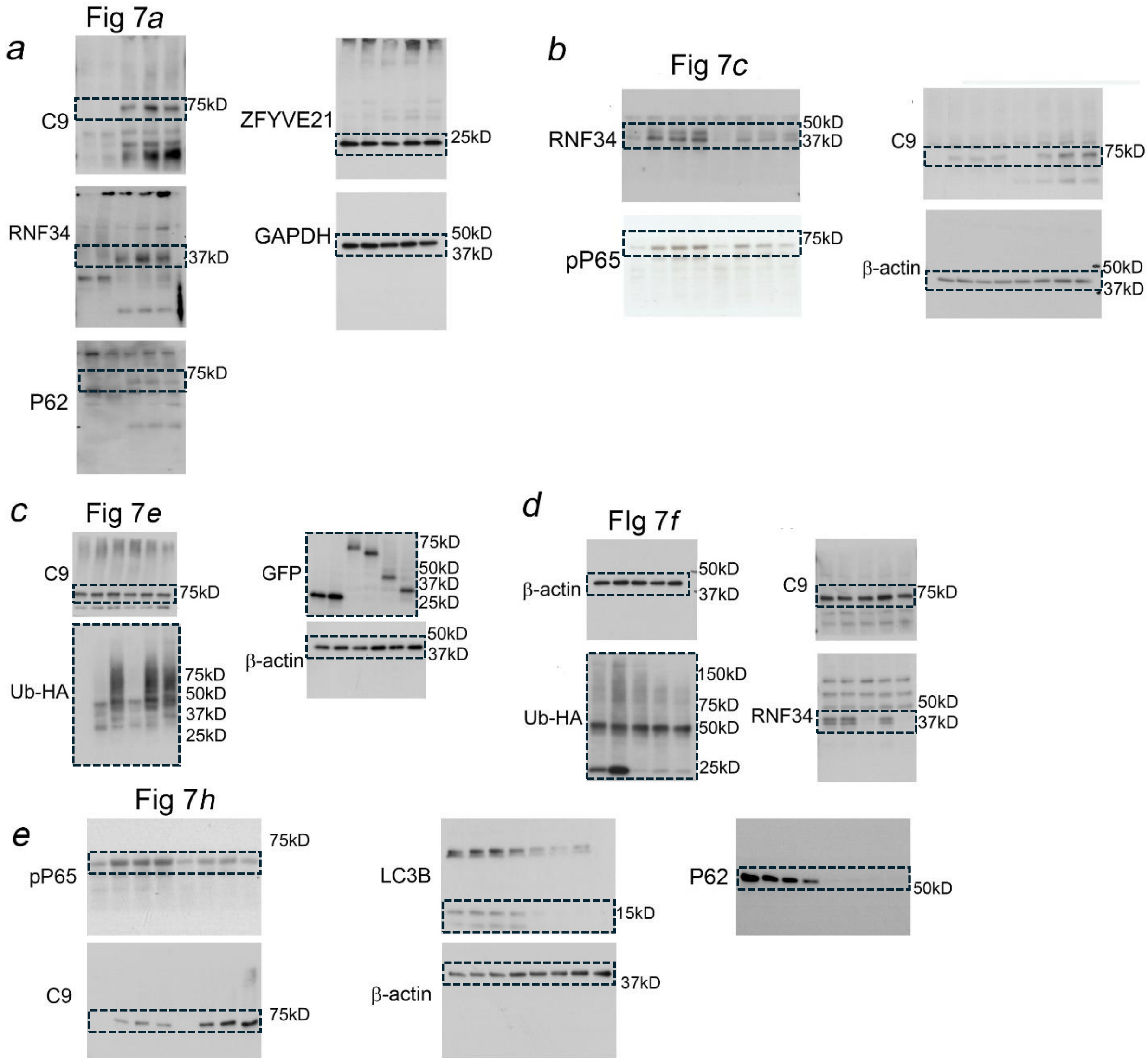

Supplementary Figure 11. Original uncropped films corresponding to Western blots in main Figure 7 in the manuscript.

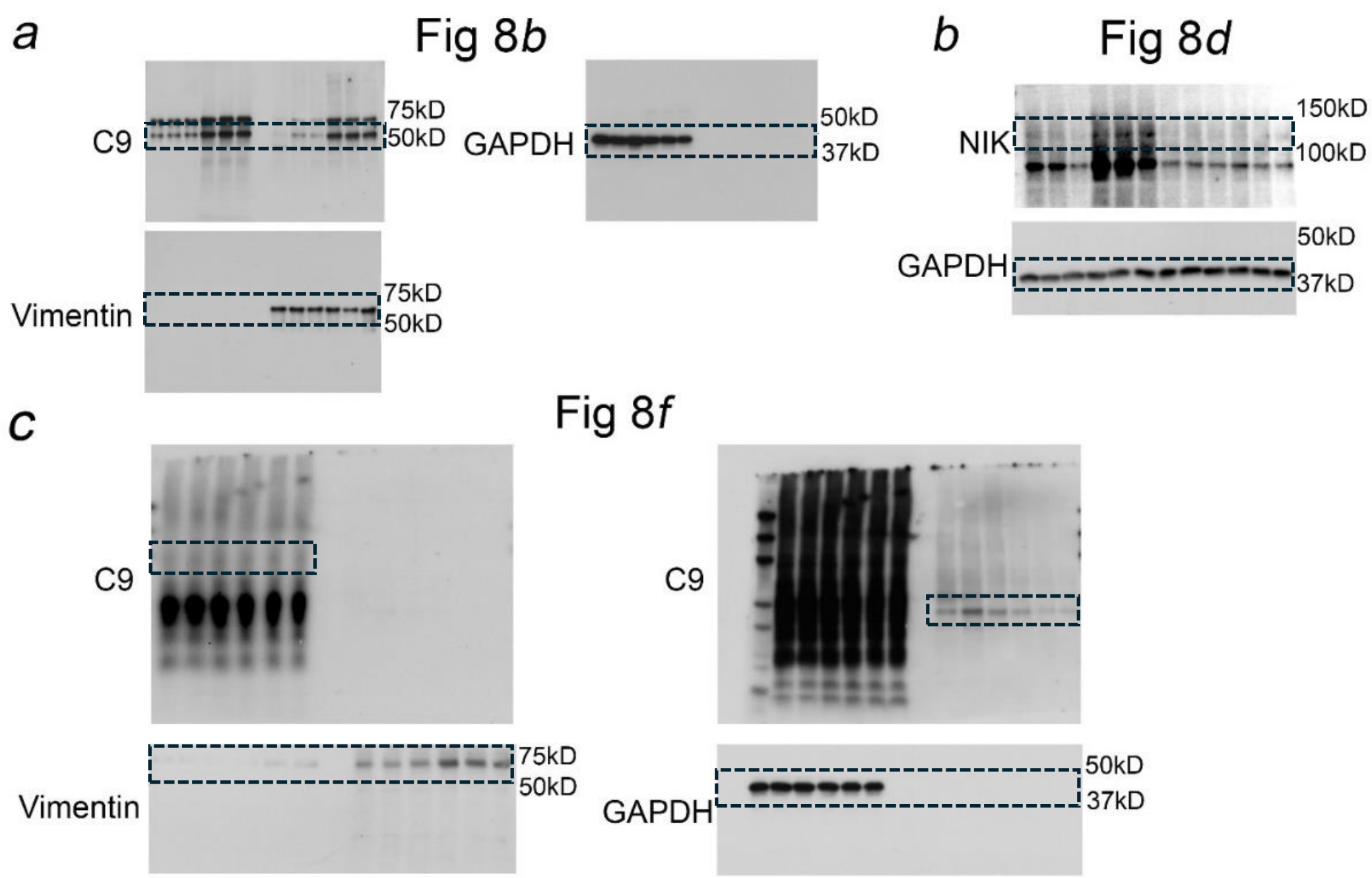

Supplementary Figure 12. Original uncropped films corresponding to Western blots in main Figure 8 in the manuscript.

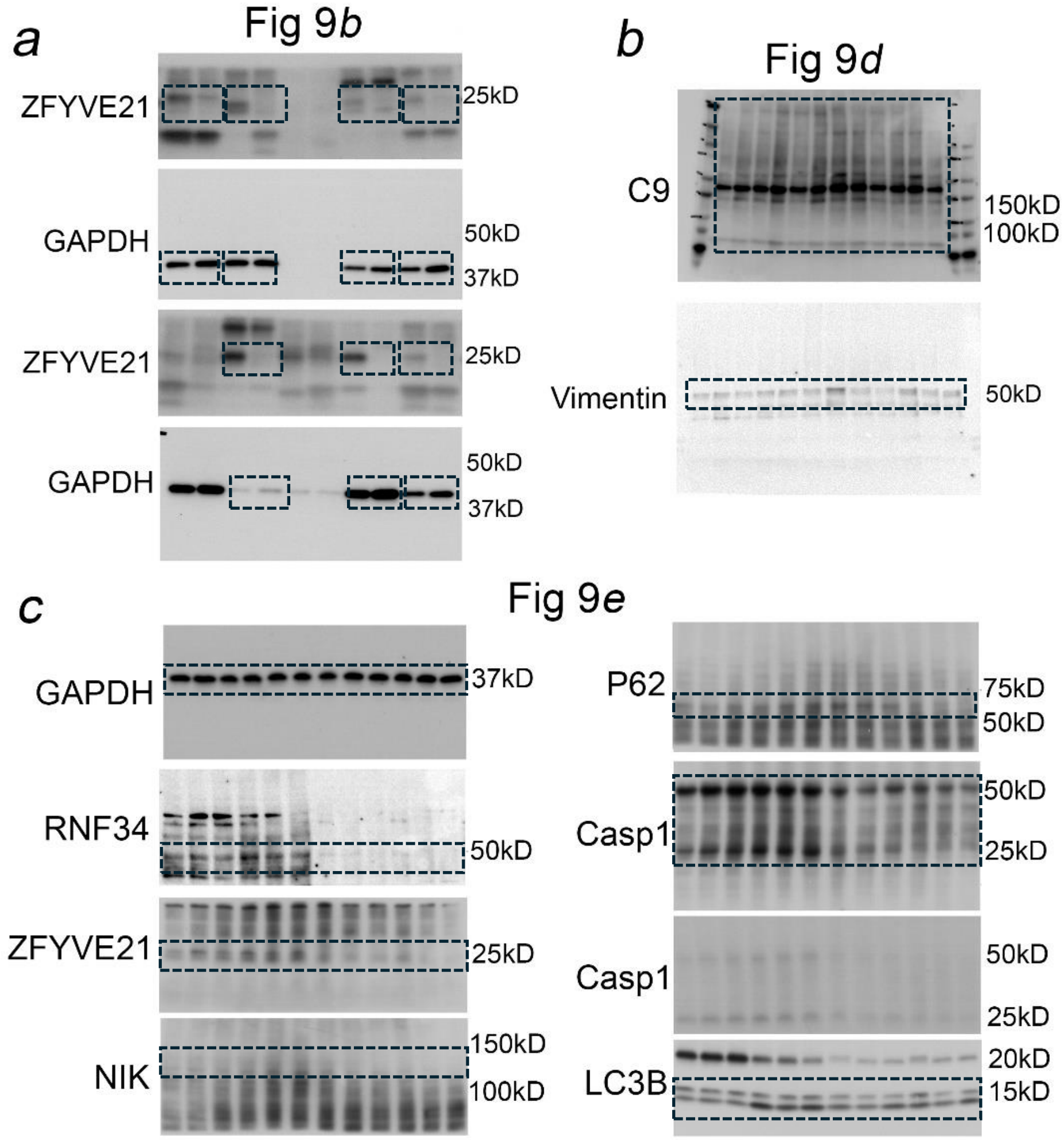

Supplementary Figure 13. Original uncropped films corresponding to Western blots in main Figure 9 in the manuscript.

SUPPLEMENTARY TABLE

Supplementary Table S1. Patient Characteristics

**CABMR**			
**Age**	**Gender**	**Race**	**Transplant**
27	F	African-American	Kidney
37	M	African-American	Heart
37	M	African-American	Heart
46	M	Caucasian	Kidney
55	M	African-American	Heart
56	M	African-American	Kidney
56	F	Caucasian	Kidney
57	M	South Asian	Kidney
60	F	Caucasian	Kidney
61	F	Caucasian	Kidney
65	F	African-American	Kidney
73	F	Caucasian	Heart
**$C4d^{+}$ ABMR**			
36	F	Caucasian	Kidney
42	M	African-American	Kidney
43	M	African-American	Kidney
52	M	African-American	Kidney
**$C4d^{-}$ ABMR**			
41	M	Caucasian	Kidney
47	M	Caucasian	Kidney
52	M	African-American	Kidney
56	M	African-American	Kidney
**TCMR**			
31	M	African-American	Kidney
42	M	Caucasian	Kidney
44	M	Hispanic	Kidney
46	M	African-American	Kidney
**Age**	**Gender**	**Race**	**Transplant**
27	F	African-American	Kidney
37	M	African-American	Heart
37	M	African-American	Heart
46	M	Caucasian	Kidney
55	M	African-American	Heart
56	M	African-American	Kidney
61	F	African-American	Kidney
73	F	Caucasian	Heart

Supplementary Table S2. Data Acquisition Parameters for cryoEM

Parameters	Positions 1 and 2	Positions 3, 4 and 5
Microscope	Titan Krios G2	Titan Krios G2
Voltage	300	300
Camera	Gatan K2	Gatan K2
Energy filter	Bioquantum	Bioquantum
Slit width (eV)	20	20
Magnification	33000	33000
Pixel Size (Å/pixel)	4.33	4.33
Does per tilt image (e/$Å^2$)	3.2	3.2
Total electron dose (e/$Å^2$)	102.4	131
Defocus range µm	7 to 9	7 to 9
Tilt range	-69 to 24	-69 to 51
Tilt increment	3°	3°
Software	Tomography 5.19	Tomography 5.19
