# Supplementary material for: Internalized components of membrane attack complexes disrupt proteostasis and acquire alarmin-like properties
Source: Nat Commun. 2026 Jul 17;17:8770. doi: 10.1038/s41467-026-75696-0 (PMC13494016; doi:10.1038/s41467-026-75696-0)
Supplement: Supplementary file 2 [file 41467_2026_75696_MOESM2_ESM.pdf]

Description of Additional Supplementary Files

Supplementary Movie 1. HUVECs stably transduced with Rab5-GFP were treated with PRA containing pHrodo-labeled C9 prior to live cell imaging.

Supplementary Movie 2. HUVECs stably transduced with ZFYVE21-GFP were treated with PRA containing AF647-labeled C9 prior to live cell imaging.
