# Supplementary material for: Internalized components of membrane attack complexes disrupt proteostasis and acquire alarmin-like properties
Source: Nat Commun. 2026 Jul 17;17:8770. doi: 10.1038/s41467-026-75696-0 (PMC13494016; doi:10.1038/s41467-026-75696-0)
Supplement: Supplementary file 3 [file 41467_2026_75696_MOESM5_ESM.pdf]

nature portfolio

Corresponding author(s): Guiyu Song, Dan Jane-wit

Last updated by author(s): 06-11-2026

Reporting Summary

Nature Portfolio wishes to improve the reproducibility of the work that we publish. This form provides structure for consistency and transparency in reporting. For further information on Nature Portfolio policies, see our Editorial Policies and the Editorial Policy Checklist.

Statistics

For all statistical analyses, confirm that the following items are present in the figure legend, table legend, main text, or Methods section.

n/a	Confirmed	
☐	☒	The exact sample size (n) for each experimental group/condition, given as a discrete number and unit of measurement
☐	☒	A statement on whether measurements were taken from distinct samples or whether the same sample was measured repeatedly
☐	☒	The statistical test(s) used AND whether they are one- or two-sided<br>*Only common tests should be described solely by name; describe more complex techniques in the Methods section.*
☐	☒	A description of all covariates tested
☐	☒	A description of any assumptions or corrections, such as tests of normality and adjustment for multiple comparisons
☐	☒	A full description of the statistical parameters including central tendency (e.g. means) or other basic estimates (e.g. regression coefficient) AND variation (e.g. standard deviation) or associated estimates of uncertainty (e.g. confidence intervals)
☐	☒	For null hypothesis testing, the test statistic (e.g. F, t, r) with confidence intervals, effect sizes, degrees of freedom and P value noted<br>*Give P values as exact values whenever suitable.*
☐	☒	For Bayesian analysis, information on the choice of priors and Markov chain Monte Carlo settings
☐	☒	For hierarchical and complex designs, identification of the appropriate level for tests and full reporting of outcomes
☐	☒	Estimates of effect sizes (e.g. Cohen's d, Pearson's r), indicating how they were calculated

Our web collection on statistics for biologists contains articles on many of the points above.

Software and code

Policy information about availability of computer code

Data collection	N/A
Data analysis	N/A

For manuscripts utilizing custom algorithms or software that are central to the research but not yet described in published literature, software must be made available to editors and reviewers. We strongly encourage code deposition in a community repository (e.g. GitHub). See the Nature Portfolio guidelines for submitting code & software for further information.

Data

Policy information about availability of data

All manuscripts must include a data availability statement. This statement should provide the following information, where applicable:

- Accession codes, unique identifiers, or web links for publicly available datasets
- A description of any restrictions on data availability
- For clinical datasets or third party data, please ensure that the statement adheres to our policy

RNA seq datasets are publicly available at the Gene Expression Omnibus (https://nam12.safelinks.protection.outlook.com/?url=https%3A%2F%2Fwww.ncbi.nlm.nih.gov%2Fgeo%2Fquery%2Facc.cgi%3Facc%3DGSE268767&data=05%7C02%7Cdan.jane-wit%40yale.edu%7C8d446904d33648b1c99508dc81785c69%7Cdd8cbebb21394df8b4114e3e87abeb5c%7C0%7C0%7C638527601156567211%7CUnknown%

7CTWFpbGZsb3d8eyJWIjoiMC4wLjAwMDAiLCJQIjoiV2luMzIiLCJBTiI6Ik1haWwiLCJXVCI6Mn0%3D%7C0%7C%7C%
7C&sdata=IluYTtIX60NhgKFNzaqGA53XHrlAjsPzjkOONErQDKk%3D&reserved=0)

Research involving human participants, their data, or biological material

Policy information about studies with human participants or human data. See also policy information about sex, gender (identity/presentation), and sexual orientation and race, ethnicity and racism.

Reporting on sex and gender	Sex was used to connote biological male and female splenocytes and allograft tissues, respectively. Sex was reported for clinical subjects in Supplementary Table 1.
Reporting on race, ethnicity, or other socially relevant groupings	Race and ethnicity for patient subjects were self-reported and listed in Supplementary Table 1.
Population characteristics	Biopsies from patients with a diagnosis of antibody-mediated rejection but not amyloidosis or post-transplant lymphoproliferative disease were recruited.
Recruitment	Patient biopsies were obtained from the Dept of Pathology at Yale University.
Ethics oversight	All protocols were approved by the Yale IACUC (#2023-20175) and IRB (#25173).

Note that full information on the approval of the study protocol must also be provided in the manuscript.

Field-specific reporting

Please select the one below that is the best fit for your research. If you are not sure, read the appropriate sections before making your selection.

☒ Life sciences ☐ Behavioural & social sciences ☐ Ecological, evolutionary & environmental sciences

For a reference copy of the document with all sections, see nature.com/documents/nr-reporting-summary-flat.pdf

Life sciences study design

All studies must disclose on these points even when the disclosure is negative.

Sample size	Sample sizes were determined to detect a simple difference of 20% between treatment groups at p<0.05 at 80% power.
Data exclusions	There were no specific instances of data exclusion.
Replication	Experiments were repeated at least 3 times using separate donors for endothelial cells. Multiple approaches were undertaken for various salient points in the study. Studies were performed in sufficiently powered mouse cohorts using genetically inbred mouse strains for reproducibility.
Randomization	As cultured endothelial cells appear morphologically identical under phase contrast, sample wells were randomly selected for interventions in vitro.
Blinding	For in vivo studies, a separate researcher blinded to the mouse genotypes and interventions performed analyses in a blinded manner.

Reporting for specific materials, systems and methods

We require information from authors about some types of materials, experimental systems and methods used in many studies. Here, indicate whether each material, system or method listed is relevant to your study. If you are not sure if a list item applies to your research, read the appropriate section before selecting a response.

Materials & experimental systems

n/a	Involved in the study	
☐	☒	Antibodies
☐	☒	Eukaryotic cell lines
☒	☐	Palaeontology and archaeology
☐	☒	Animals and other organisms
☒	☐	Clinical data
☒	☐	Dual use research of concern
☒	☐	Plants

Methods

n/a	Involved in the study	
☒	☐	ChIP-seq
☒	☐	Flow cytometry
☒	☐	MRI-based neuroimaging

Antibodies

Antibodies used	Antibodies used in the study are described in the Materials & Methods section.
Validation	Antibodies were validated by the commercial vendor and by other research groups. Antibody specificity was tested using siRNA against the relevant target molecule.

Eukaryotic cell lines

Policy information about cell lines and Sex and Gender in Research

Cell line source(s)	HEK293 cells were purchased and validated from ATCC. Human Umbilical Vein Endothelial Cells (HUVECs) were purchased from the Yale Vascular Biology & Therapeutics core service.
Authentication	Identity of HUVECs were confirmed by flow cytometry for CD31 prior to use.
Mycoplasma contamination	Cells were tested for Mycoplasma contamination once a month.
Commonly misidentified lines (See ICLAC register)	N/A

Animals and other research organisms

Policy information about studies involving animals; ARRIVE guidelines recommended for reporting animal research, and Sex and Gender in Research

Laboratory animals	Mus musculus (C57/Bl6, H2b)
Wild animals	N/A
Reporting on sex	Male skin allografts were used as these tissues express the H-Y minor mismatch alloantigen which is immunologically targeted by male splenocytes. This was reported in the Materials & Methods section.
Field-collected samples	N/A
Ethics oversight	All protocols were approved by the Yale IACUC (#2023-20175) and IRB (#25173).

Note that full information on the approval of the study protocol must also be provided in the manuscript.

Plants

Seed stocks	N/A
Novel plant genotypes	N/A
Authentication	N/A
