# Supplementary material for: Internalized components of membrane attack complexes disrupt proteostasis and acquire alarmin-like properties
Source: Nat Commun. 2026 Jul 17;17:8770. doi: 10.1038/s41467-026-75696-0 (PMC13494016; doi:10.1038/s41467-026-75696-0)
Supplement: Supplementary file 4 [file 41467_2026_75696_MOESM6_ESM.pdf]

nature portfolio

Peer Review File

Internalized Components of Membrane Attack Complexes Disrupt Proteostasis and Acquire Alarmin-Like Properties

Corresponding Author: Dr Dan Jane-wit

Parts of this Peer Review File have been redacted as indicated to remove third-party material.

This file contains all reviewer reports in order by version, followed by all author rebuttals in order by version.

Version 0:

Reviewer comments:

Reviewer #1

(Remarks to the Author)
Dan Jane-wit and colleagues have provided strong molecular evidence that Membrane Attack Complexes (MACs) are internalized in Rab5+ vesicles to promote aggrephagy that results in NF-KB activation to promote endothelia cell activation. These particular studies are well done and add to or support their past work, particulary Fang et al. 2019. Nat. Comm. and Fan et al. 2023. Nat. Comm. The concept that DSA induced MACS functions as alarmins is novel. All sections of the manuscript are well written, including the methods. I I do have some concerns that, if addressed, would add to the robustness and impact of the work.

1. At the present, the impact of the examined pathway (Donor Specific Antibody -> MACs -> Rab5+ vesicles -> Aggregates -> Aggrephagy -> NF-kB -> Endothelial cell (EC) activation) on innate or adaptive immunity and graft outcome is poorly explored. This is necessary to support the suggestion that MACs act as an alarmin. To meet the definition of an alarmin as outlined by Oppenheim, MACs would be released from their sequestered state in EC and recognized by recipient immune cells. The data presented supports that MACs trigger an inflammatory process involving aggrephagy and causing EC activation that results in local inflammation. Thus the suggestion that MACs are alarmins is not supported. This could be improved by characterization of the adoptively transferred recipient immune response. Are there differences in T cell responses to the allograft locally or systemically? Is there differences in myeloid cell activation (the SCID mice should have myeloid cells to assess). More investigations into how disruption of the examined pathway shapes innate and adaptive immune responses and outcomes are needed. It also seems like the anti-H-2b ab treated donor organs (heart/kidney), as well as skin could be transplanted into WT mice to mice to get an appreciation for how recipient immune response are influenced by the proposed MAC alarmin activity. The authors need to distinguish this MAC alarmin activity is distinct from general inflammation due to inflammatory cell death of EC or NF-Kb activation of EC.

2. The data in figure 1 is not overly convincing. Much of the data lack quantification and the data are a very limited assessment of biopsies with chronic antibody mediated rejection or no rejection. Adding more samples (CABMR and control) as well as other relevant groups that would be expected to have less MACS/protein aggregates groups (acute and chronic TCMR versus C4d+ ABMR, C4d- ABMR, or CABMR) would better support the conclusion that DSA triggers this alarmin function of MACs.

Reviewer #2

(Remarks to the Author)
This study investigates the formation and role of membrane attack complexes (MACs) and their component C9 in endothelial cells, with a special focus on NFkappaB activation. The authors first investigated tissue samples of chronic antibody mediated rejection (CABMR) patients and found elevated levels of thioflavin stainings, reporting protein aggregates, in C9-positive cells. Using panel reactive antibody (PRA) sera-stimulated HUVEC, these findings were recapitulated. Moreover, C9 was found to form aggregates in these cells. Subcloning and overexpression experiments showed that the region aa197-270 of C9 is responsible for the formation of aggregates. Use of inhibitors of clathrin-mediated endocytosis such as dynasore, or overexpression of dominant negative Rab5, further showed that most of these aggregates form intracellularly, subsequent to internalization and acidification, most likely in vesicles of the endolysosomal pathway. Further experiments

showed that C9 becomes ubiquitinylated upon PRA serum activation, and increasingly associated with autophagic markers such as LC3B. Furthermore, siRNA-mediated depletion of autophagic regulators such as ATG5 led to increased levels of C9. Similar results were obtained for overexpressed aa197-270. This overexpression also led to increased levels of NFkappaB activation, which could again be reduced by ATG5 depletion. The authors further show that the Rab5-associated protein ZFYVE21 is upregulated in PRA-stimulated HUVEC and colocalized with C9 positive vesicles. SiRNA-mediated depletion of ZFYVE21 led to elevated levels of C9 and reduced autophagosome formation. Co-IPs showed interaction of ZFYVE21 with LC3A and LC3B, which is abrogated by mutation of the critical L60, Y146, or L149 residues of ZFYVE21. Proteomic analyses of the ZFYVE21 interactome in PRA-stimulated cells identified several proteins containing E3 ubiquitin ligase activity, among them RNF34. RNF34 was found to colocalize with ZFYVE21-positive, thioflavin stained dots, while siRNA mediated depletion of RNF34 reduced C9 ubiquitinylation, enhanced C9 levels, and attenuated NFkappaB signalling. P62, a ubiquitin binding protein, co-IPed and colocalized with ZFYVE21-positive puncta that stained for thioflavin, while its siRNA-mediated depletion led to decreased NFkappaB activity. The authors then tested the identified signalling pathways in vivo using mouse models, such as a humanized model of ischemia reperfusion injury, leading to complement activation and MAC formation. C9 intracellular aggregates were also detected in this model, while C3-/- mice, lacking the ability to form MACs, showed decreased thioflavin staining and reduced NFkappaB activity. C9 aggregation was also reduced by Dynasore treatment. Furthermore, ZFYVE21-/- mice shoed increased thioflavin staining and elevated levels of insoluble C9, strongly reduced expression of RNF34 and reduced NFkappaB activation. Implanted HUVEC microvessels treated with RNF34 shRNA also showed reduced C9 agregation. Finally, using a skin allograft model, H2b antibody-stimulated wt skin grafts showed increased epidermal thickening and immune cell infiltration, which was reduced in ZFYVE21endothlial -/- mice and mice globally lacking ZFYVE21, while ZFYVE21 EC -/- skin grafts showed decreased levels of cytokines and chemokines.

This study assembles a wealth of data and experimental approaches to make a case for a non-cytolytic role of the MAC component C9, the formation of respective aggregates, and its association with ZFYVE21 and RNF34-P62 complexes in NFkappaB activation in endothelial cells in vitro and in vivo. It is very densely written, and experiments are sometimes explained or interpreted only sparingly, which restricts its accessibility.

Major points:
1) In the micrographs presented, it is often hard to appreciate colocalization of stainings such as thioflavin or ZFYVE21 with other cellular markers. Please provide also individual channels for thioflavin, e.g. in Fig. 2e, 3g, or ZFYVE21 in Fig. 6c, 7j.
2) Fig. 3g: The authors use overexpression of a dominant negative mutant of Rab5 to demonstrate presence of C9 in the endolysosomal pathway. How were cells expressing this construct identified?. Please also specifiy the isoform that was used (a, b, or c?). Also, as RabGTPases share many GAPs and GEFs, a more convincing experiment would be siRNA-mediated depletion of Rab5(a).
3) Related to 2): To really show the presence of C9 within endosomes, overexpression or staining of endogenous Rab5(a) or EEA1 should be used.
4) lines 165-170, Fig. 3h. The authors state that “C9 aggregates form in a manner requiring…endosome acidification”. As interference with endosome acidification using bafilomycin or NH4Cl reduced aggregation by only 30%, this statement should be amended.
5) lines 178,179, Fig. 4d,e,g: “…C9 became containing within LC3+ membranes…displaying markers specific for aggresomes.” Two separate stainings, one for LC3-RFP, and one for HSP70, are shown. It is thus not clear whether LC3-RFP membranes also display HSP70.
6) lines 183,184: Fig. 4k: “AA197-270…colocalized with Thioflavin+Hsp70+aggresomes”. This colocalization appears to be only partial. Please provide a quantification for this colocalization using a statistically significant number of cells.
7) lines 248-265: In some cases, the results of specific experiments seem like a foregone conclusion. For example, the authors perform a proteomic analysis, looking for EB3 ubiquitin ligases binding to ZFYVE21, only to pick RNF34, a protein that they already identified as a ZFYVE21 binding protein in a previous publication (Li et al., Nat Commun, 2023).
8) Fig. 6g: Please explain what is shown here in more detail. The legend is not really helpful.
9) Fig. 9j: the labelling under the bar diagram is probably not correct. Please check.
10) Discussion: Given the wealth of data presented and their potential implications, the discussion appears to be rather sparse.

Minor points:
1) abstract and text: “...promote NFkappaB…” please add “activity”.
2) lines 176,177: “In co-immunoprecipitations…C9 became ubiquitinylated”. Please repharse, as C9 is already ubiquitinylated before the co-IP.
3) line 223: “became upregulated…”. Here, probably “ZFYVE21” is missing.
4) E.g. Fig. 3g: It is sometimes not clear what individual dots in diagrams represent, aggregates, cells or donors? Please add this information to the figure legends.
4) Fig. 5j: The results showing CXCL7 and CXCL11 levels are not mentioned in the text.

Reviewer #3

(Remarks to the Author)
The manuscript by Song et al investigates the intracellular fate of complement membrane attack complex (MAC). In the manuscript the authors show that C9, a MAC protein, promotes loss of proteostasis to become an intracellular “alarmin”. The authors show that C9 is internalised into Rab5 endosomes, promoting C9 aggregation and inducing NF-kB activation. This process seems to require ZFYVE21 , a Rab5 effector that links LC3 to RNF34-P62 complexes to mediate C9 aggrephagy.

The authors confirm the involvement of ZFYVE21 by using gene deficient mice lacking ZFYVE21, that show reduced MAC-induced tissue injury in a skin model of chronic rejection.
Overall, the manuscript is interesting, but the only novel element is the possible involvement of ZFYVE21 and its involvement is not very convincing. The senior author has previously demonstrated the internalisation of MAC via Rab5+ endosomes and the triggering of pro-inflammatory cytokines via the NF-kB pathway (PNAS 2015, 112(31):9689-9691, doi: 10.1073/pnas.1503535112). In this study, they demonstrate the C9 internalisation via Rab5 and then claim that C9 polymerises intracellularly although I am not convinced by the data shown. C9 is shown to aggregate in cell free solution at lower pH but that does not demonstrate that this aggregation occurs intracellularly and we don't actually have the whole MAC (C9 already aggregated) internalised into Rab5+ endosomes. The authors keep using Thioflavin as a stain for aggregated protein, but the specificity of thioflavin is also questionable. Thioflavin and Congo Red are histological stains that have traditionally been used to stain b-pleated sheets of amyloid. Their use in this manuscript to demonstrate aggregated protein raises questions regarding specificity – what exactly are the authors staining for when they see positive thioflavin staining? Having said that, I also feel that the quality of the images is poor and does not clearly show what the authors want to show. In order to properly visualise C9 aggregation in intracellular organelles high resolution fluorescent microscopy should be employed. Also a time-lapse video of fluorescently labelled C9 would be very helpful in this case, so that the authors can demonstrate internalisation of C9 through Rab5, aggregation (which can be determined by the increase in fluorescent intensity) and then interaction with ZFYVE21??? Biochemical analysis showing C9 aggregation in Rab5+ organelles is also essential to convince this reviewer. The authors should isolate the organelle fraction using differential centrifugation and biochemically analyse the contents of the organelles at different time points (i.e. look for C9, Rab5, ZFYVE21, LC3, etc).
The only novel element of the manuscript is the possible involvement of ZFYVE21 and again I don't find the data presented particularly convincing. The authors do utilise mouse models to demonstrate this, although I think CRISPR knockout of several components of this pathway (Rab5, ZFYVE21, LC3 etc) on HUVECs would have been much better. They claim that ZFYVE21 deficient animals demonstrate a reduced MAC-induced tissue injury in a skin model. I fail to see how they can make that claim when the tissue injury in this skin model can not be just MAC-induced but must involved other immunological elements.
An interesting paper, confirming some of the author's previous findings, but lacking the evidence for the novel part of the claims, regarding the involvement of ZFYVE21 and autophagy.

Version 1:

Reviewer comments:

Reviewer #1

(Remarks to the Author)
This manuscript is much improved and represents a tour de force investigation of non-cytolytic immunogenicity of MAC. The data are robust and span both in vitro and precise in vivo studies that clearly dictate the immune cells involved in non-cytolytic functions of MACs and the molecular players completing the steps to activate the C9 based aggnephagy leading to NF-KB signaling and EC activation.

The authors have more than adequately addressed my prior concerns around innate and adaptive immunity in the C9/MAC driven inflammation. The additional studies of clinical samples in Figure 1 are noted. Likewise, new mechanistic data all contribute to a complete and interesting story that opens up new avenues for therapeutics to address antibody mediated pathologies in transplantation and potentially other antibody based immunopathologies.

I commend the others on their study.

Reviewer #2

(Remarks to the Author)
The authors have addressed all of my raised points sufficiently.

A small detail regarding my major point 9):
"Fig. 9j: the labelling under the bar diagram is probably not correct. Please check."
This included a typo, actually meaning Fig. 8j. Please check the labelling there.
This is a correction that I don´t need to see.

Reviewer #3

(Remarks to the Author)
I feel that the re-submitted manuscript by Dan Jane-wit and colleagues is much improved from the original submission and the authors have made an effort to address all the reviewers' comments.

They have provided evidence that Membrane Attack Complexes (MACs) are internalized in Rab5+ vesicles to promote aggrephagy that results in NF-KB activation. These findings have been described in their past work, Fang et al. 2019. Nat.

Comm. and Li et al. 2023. Nat. Comm. thus I have been struggling with the novelty of the findings. The involvement of ZFYVE21 is a novel element and the authors have taken the comments on board to strengthen that finding with CRISPR knockouts.

In addition, the authors have made an attempt to generate time-lapse video of fluorescently labelled C9 to demonstrate internalisation of C9 through Rab5. I do not find the actual videos convincing - but the stills in the figures seem to demonstrate the point. I think the authors need to quantify the co-localisation in those images in order to strengthen their point of C9 internalisation in Rab5 endosomes. Furthermore, as I have previously suggested it would be good to also show the interaction (co-localisation) with ZFYVE21 given that this one of the most novel findings of the work.

Overall an improvement to the original submission and an honest attempt to address all of the reviewers' comments.

Version 2:

Reviewer comments:

Reviewer #2

(Remarks to the Author)
I am very satisfied with the current version of the manuscript and recommend acceptance.

Reviewer #3

(Remarks to the Author)
I feel that the re-submitted manuscript by Dan Jane-wit and colleagues is much improved from the original submission and the authors have made an effort to address all the reviewers' comments.
Although I had previously struggled to see the novelty of their findings, in the current version the authors have gone to great lengths to stress the novelty. The introduction is almost entirely re-written to present the new findings. They have identified NUMBL as a Rab35 effector that binds to C9 and promotes its internalisation. Once in endolysosomes, C9 aggregates are targeted for aggrephagy in a process that activates NF-kB. The importance of ZFYVE21 and RNF34 in this process is highly novel. The authors also use mice in order to detect these C9 aggregates in vivo. The new version of the manuscript clearly demonstrates the novelty of the findings demonstrating immunogenic MAC aggregates promoting aggrephagy I feel that the extensive revisions have alleviated prior concerns that I had regarding novelty, and that this manuscript is now suitable for publication to Nature Communications

Open Access This Peer Review File is licensed under a Creative Commons Attribution 4.0 International License, which permits use, sharing, adaptation, distribution and reproduction in any medium or format, as long as you give appropriate credit to the original author(s) and the source, provide a link to the Creative Commons license, and indicate if changes were made.
In cases where reviewers are anonymous, credit should be given to 'Anonymous Referee' and the source.
The images or other third party material in this Peer Review File are included in the article’s Creative Commons license,

unless indicated otherwise in a credit line to the material. If material is not included in the article's Creative Commons license and your intended use is not permitted by statutory regulation or exceeds the permitted use, you will need to obtain permission directly from the copyright holder.
To view a copy of this license, visit https://creativecommons.org/licenses/by/4.0/

02/010/2025

Reviewers, Nature Communication:

This letter provides point-by-point revisions to address concerns from 3 separate reviewer comments. We are appreciative of the opportunity to submit our work to Nature Communications, and we are thankful for the reviewer comments which we believe have improved the quality of the manuscript. We apologize for this delayed resubmission due to delays in prospective enrollment for new tissue sections and breeding studies for a new mouse strain.

REVIEWER COMMENTS

Reviewer #1 (Remarks to the Author):

Dan Jane-wit and colleagues have provided strong molecular evidence that Membrane Attack Complexes (MACs) are internalized in Rab5+ vesicles to promote aggrephagy that results in NF-KB activation to promote endothelia cell activation. These particular studies are well done and add to or support their past work, particularly Fang et al. 2019. Nat. Comm. and Li et al. 2023. Nat. Comm. The concept that DSA induced MACS functions as alarmins is novel. All sections of the manuscript are well written, including the methods. I do have some concerns that, if addressed, would add to the robustness and impact of the work. We thank the reviewer for these comments.

1. At the present, the impact of the examined pathway (Donor Specific Antibody -> MACs -> Rab5+ vesicles -> Aggregates -> Aggrephagy -> NF-kB -> Endothelial cell (EC) activation) on innate or adaptive immunity and graft outcome is poorly explored. This is necessary to support the suggestion that MACs act as an alarmin. To meet the definition of an alarmin as outlined by Oppenheim, MACs would be released from their sequestered state in EC and recognized by recipient immune cells. The data presented supports that MACs trigger an inflammatory process involving aggrephagy and causing EC activation that results in local inflammation. Thus the suggestion that MACs are alarmins is not supported. In acknowledgement of the definition of alarmins as outlined by the R1::

1. We changed the title of the manuscript to reflect our belief that C9 aggregates show ‘alarmin-like’ properties. The new title of the paper is, ‘Complement Membrane Attack Complexes Disrupt Proteostasis to Acquire Alarmin-Like Properties.’

2. Clarification into alarmin-like behavior of C9 aggregates is provided in the Discussion section:

Classically, alarmins connote intracellularly sequestered proteins like S100 or histones that are basally non-immunogenic but that acquire immunogenicity upon being externalized and sensed by recipient immune cells. Here, we alternatively posit that C9, a protein 'sequestered' in extracellular space where it is non-immunogenic, acquires alarmin-like properties when internalized in ECs. In this process, the culprit damage-associated protein, C9, loses its homeostatic sequestration, becomes pathologically modified, and elicits immune responses within the same cell.

1. Clarification into alarmin-like behavior of C9 aggregates is provided in the Introduction section:

Alarmins are endogenous proteins that are basally non-immunogenic but become pro-inflammatory after losing their homeostatic compartmentalization[28] or after becoming pathologically modified as is the case for amyloidogenic proteins.[29] Based on the aggregate-prone features of C9 and the spatially restricted responses of MACs, we tested the hypothesis that intracellular C9 forms aggregates that operationally behave similar to alarmins.

More investigations into how disruption of the examined pathway shapes innate and adaptive immune responses and outcomes are needed. This is important to allow an appreciation for how recipient immune response are influenced by the proposed MAC alarmin activity. This could be improved by characterization of the adoptively transferred recipient immune response. Are there differences in T cell responses to the allograft locally or systemically?

We believe that this is a valuable comment, and we performed new experiments as above to obtain deeper insights into the immunostimulatory effects of MACs:

1. We treated WT C57/Bl6 (H-2^b) skin graft with anti-$H2^b$ to form MACs on ECs. We then transplanted MAC-treated skin ton C57/Bl6 mice with both lymphoid and myeloid cells, SCID/bg mice with myeloid cells only, and SCID/bg mice treated with liposomal clodronate and having no lymphoid or myeloid cells. Using this model, in Figure 10*a,b* we found that both lymphoid and myeloid cells contributed to the ability of MACs to promote tissue injury and tissue inflammation (Figure 10*a,b*).

2. Using the model above, we secondly found that lymphoid cells and MACs showed ~50% contributions towards EC activation, defined as upregulated transcripts of EC-specific markers of EC activation including VCAM1 and SELE (E-selectin) in Figure 10*c*.

3. In a second *in vivo* study, as per R1's suggestions, we comprehensively phenotyped the immune response of passively transferred splenocytes across the tissue compartments including skin, blood, and spleen. We treated male C57/Bl6 (H-2^b) or male ZFYVE21 $EC^{-/-}$ skin grafts with anti-$H2^b$ to form MACs on ECs, and we placed Ab-treated skin grafts onto SCID/bg hosts passively received female splenocytes. Female splenocytes show a minor H-Y alloantigen mismatch to male skin grafts and mount a chronic inflammatory response.

   We then analyzed frequencies and activation markers for lymphoid cells (CD4+ T cells, CD8+ T cells) and myeloid cells (CD11b+ cells, NK1.1+ cells). We found that MAC-treated ECs systemically expanded and activated both lymphoid and myeloid cells in the skin, blood, and spleen, indicating systemic effects of MACs (Figure 10*g,h*).

4. In the experiment above, we secondly found that ZFYVE21 in ECs strongly reduced lymphoid cell infiltration and activation in the skin, demonstrating a local effect of EC-specific ZFYVE21 on lymphoid cells within peripheral tissues (Figure 10*g,h*).

5. In a third study, we treated male C57/Bl6 (H-2^b), ZFYVE21$^{-/-}$ skin grafts, and ZFYVE21 EC$^{-/-}$ skin grafts with anti-H2^b to form MACs on ECs, and we placed Ab-treated skin grafts onto SCID/bg hosts passively received female splenocytes. We found that genetic loss of ZFYVE21 in ECs and not non-ECs most strongly contributed to the ability of MACs to promote tissue injury and inflammation.

Are there differences in myeloid cell activation (the SCID mice should have myeloid cells to assess)? We performed EC:PBMC coculture, and found that MAC-treated ECs showed nonspecific immunostimulatory properties affecting both lymphoid and myeloid cells (Supplementary Figure 2*l*).These data aligned with those found *in vivo* as described above in Figure 10*g,h* as well as our studies originally describing use of PRA where we found that PRA-treated human ECs potentiated infiltration of both myeloid and lymphoid cells into human artery xenografts surgically implanted into descending aortae of SCID/bg mice engrafted with human PBMCs *in vivo* (PMCID: PMC3885874).

[REDACTED]

The authors need to distinguish how MAC alarmin activity is distinct from general inflammation due to inflammatory cell death of EC or NF-Kb activation of EC. This is an important point. In recombination studies, PRA sera was fractionated into IgG+ fraction which, when combined with C9-deficient reference sera, almost completely lost the ability to induce NF-κB genes in qRT-PCR studies. Here, differences in threshold cycles of x indicates decrease in 2^x transcript expression, meaning that for these NF-κB genes, loss of C9 reduced NF-κB transcripts by an estimated $\geq 2^4$. This indicated that NF-κB activity induced by IgG or anaphylatoxins (C3a, C5a) showed relatively minor contributions towards NF-κB compared to C9's alarmin-like activity (PMCID: PMC3885874).

[REDACTED]

Unlike xenogeneic bacteria or anucleated RBCs, we did not observe significant osmolysis of nucleated HUVECs with PRA treatment. To induce PRA, we use a buffer called gelatin veronal buffer which in itself is toxic to cells after cultures for >8 hrs. In our original studies (PMCID: PMC3885874), we treated HUVECs with control 'non-PRA' sera and PRA sera and found no differences in cell death at 4 hrs. We repeated these EC death studies in Figure 2a at earlier times (1, 2, and 3hrs) and similarly we did not observe increased EC death for the PRA treatment durations used throughout the present manuscript, thereby ruling out MAC-induced osmolysis as a cause for increased NF-κB.

2. The data in figure 1 is not overly convincing. Much of the data lack quantification and the data are a very limited assessment of biopsies with chronic antibody mediated rejection or no rejection. Adding more samples (CABMR and control) as well as other relevant groups that would be expected to have less MACS/protein aggregates groups (acute and chronic TCMR versus C4d+ ABMR, C4d- ABMR, or CABMR) would better support the conclusion that DSA triggers this alarmin function of MACs. To address the reviewer's comments, we have obtained additional CABMR (n=12 total), additional controls

(n=4) as well as various rejection subtypes including TCMR (n=4), C4d+ ABMR (n=4), and C4d- ABMR (n=4), and renal cell carcinoma (n=4) biopsies for analysis. We feel that this expanded analysis was very informative in that histological evidence of thioflavin staining was predominantly observed in conditions involving abundant MAC formation, namely chronic ABMR, C4d+ ABMR, and renal cell carcinoma but not TCMR or C4d- ABMR. These analyses incorporated statistical quantifications for EC activation (VCAM-1) and EC death (TUNEL). Our data suggested that conditions involving persistent or ongoing MAC assembly are more likely to cause disrupted proteostasis and formation of C9 aggregates.

Reviewer #2 (Remarks to the Author):

This study investigates the formation and role of membrane attack complexes (MACs) and their component C9 in endothelial cells, with a special focus on NFkappaB activation. The authors first investigated tissue samples of chronic antibody mediated rejection (CABMR) patients and found elevated levels of thioflavin stainings, reporting protein aggregates, in C9-positive cells. Using panel reactive antibody (PRA) sera-stimulated HUVEC, these findings were recapitulated. Moreover, C9 was found to form aggregates in these cells. Subcloning and overexpression experiments showed that the region aa197-270 of C9 is responsible for the formation of aggregates. Use of inhibitors of clathrin-mediated endocytosis such as dynasore, or overexpression of dominant negative Rab5, further showed that most of these aggregates form intracellularly, subsequent to internalization and acidification, most likely in vesicles of the endolysosomal pathway. Further experiments showed that C9 becomes ubiquitinylated upon PRA serum activation, and increasingly associated with autophagic markers such as LC3B. Furthermore, siRNA-mediated depletion of autophagic regulators such as ATG5 led to increased levels of C9. Similar results were obtained for overexpressed aa197-270. This overexpression also led to increased levels of NFkappaB activation, which could again be reduced by ATG5 depletion. The authors further show that the Rab5-associated protein ZFYVE21 is upregulated in PRA-stimulated HUVEC and colocalized with C9 positive vesicles. SiRNA-mediated depletion of ZFYVE21 led to elevated levels of C9 and reduced autophagosome formation. Co-IPs showed interaction of ZFYVE21 with LC3A and LC3B, which is abrogated by mutation of the critical L60, Y146, or L149 residues of ZFYVE21. Proteomic analyses of the ZFYVE21 interactome in PRA-stimulated cells identified several proteins containing E3 ubiquitin ligase activity, among them RNF34. RNF34 was found to colocalize with ZFYVE21-positive, thioflavin stained dots, while siRNA mediated depletion of RNF34 reduced C9 ubiquitinylation, enhanced C9 levels, and attenuated NFkappaB signaling. P62, a ubiquitin binding protein, co-IPed and colocalized with ZFYVE21-positive puncta that stained for thioflavin, while its siRNA-mediated depletion led to decreased NFkappaB activity. The authors then tested the identified signalling pathways in vivo using mouse models, such as a humanized model of ischemia reperfusion injury, leading to complement activation and MAC formation. C9 intracellular aggregates were also detected in this model, while C3-/- mice, lacking the ability to form MACs, showed decreased thioflavin staining and reduced NFkappaB activity. C9 aggregation was also reduced by Dynasore treatment. Furthermore, ZFYVE21-/- mice shoed increased thioflavin staining and elevated levels of insoluble C9, strongly reduced expression of RNF34 and reduced NFkappaB activation. Implanted HUVEC microvessels treated with RNF34 shRNA also showed reduced C9 agregation. Finally, using a skin allograft model, H2b antibody-stimulated wt skin grafts showed increased epidermal thickening and immune cell infiltration, which was reduced in ZFYVE21endothlial -/- mice and mice globally lacking ZFYVE21, while ZFYVE21 EC -/- skin grafts showed decreased levels of cytokines and chemokines. This is a very detailed summary of the salient findings of the manuscript. We thank the reviewer for careful consideration of the manuscript.

This study assembles a wealth of data and experimental approaches to make a case for a non-cytolytic role of the MAC component C9, the formation of respective aggregates, and its association with ZFYVE21 and RNF34-P62 complexes in NFkappaB activation in endothelial cells in vitro and in vivo. It is

very densely written, and experiments are sometimes explained or interpreted only sparingly, which restricts its accessibility. We thank the reviewer for the very careful and detailed review of the manuscript. The manuscript has been extensively re-written as to improve clarity and accessibility. The Introduction and Discussion sections have been extensively re-written. We believe the quality of the manuscript as well as its readability have been improved by the reviewer's comments. As above, we wish to revise our prior conclusion to now state that C9 aggregates exhibit 'alarmin-like' behavior.

Major points:
1) In the micrographs presented, it is often hard to appreciate colocalization of stainings such as thioflavin or ZFYVE21 with other cellular markers. Please provide also individual channels for thioflavin, e.g. in Fig. 2e, 3g, or ZFYVE21 in Fig. 6c, 7j. This was changed per the reviewer's request in new Supplementary Figure 1*c*, updated Figure 3*g,* Figure 6*c,* and Figure 7*j*. In these updated figures, we have increased the resolution and have enlarged the photomicrographs to better enable appreciation of colocalization of I.F. stains, and we inserted insets for select figures to further improve visualization of I.F. stains.

2) Fig. 3g: The authors use overexpression of a dominant negative mutant of Rab5 to demonstrate presence of C9 in the endolysosomal pathway. How were cells expressing this construct identified?. Please also specifiy the isoform that was used (a, b, or c?). Also, as RabGTPases share many GAPs and GEFs, a more convincing experiment would be siRNA-mediated depletion of Rab5(a). We agree with the reviewer regarding siRNA knockdowns of Rab5. However, Rab5 carries 3 isoforms, Rab5a/b/c, and we have attempted several commercial pools of siRNA (Dharmacon, ThermoFisher, Santa Cruz) to deplete Rab5a. Despite significant knockdowns for Rab5a, we have not observed biochemical or functional changes to PRA-treated HUVECs with respect to ZFYVE21 induction of NF-κB, suggesting compensatory roles for Rab5a's other isoforms, Rab5b and Rab5c. As such, we have utilized Rab5-WT and Rab5 dominant negative (DN) constructs carrying and S43N mutation where Rab5 is 'locked' into an inactive isoform where it constitutively binds Rab5-GDP but not Rab5-GTP. We have Rab5 WT and Rab5 DN constructs that all utilize the Rab5a isoform with either GFP or RFP reporters, and thus cells expressing these constructs can visualized by I.F. In our prior studies, we used the Rab5 DN construct to ablate Rab5 activity as measured by Rab5-GTP pulldowns which we were not able to do using Rab5a siRNA (PMCID: PMC11343665).

3) Related to 2): To really show the presence of C9 within endosomes, overexpression or staining of endogenous Rab5(a) or EEA1 should be used. The reviewer raises a good point, and we have performed new experiments in Figure 3*i* using an anti-Rab5 Ab (Cell Signaling, #3547) and Figure 3*j* staining for EEA1 via vesicular flow cytometry of EEA1 (Cell Signaling, #2411). In these studies, we find that the bulk of C9+Thioflavin+ signal occurs within Rab7 endosomes and appears to decrease in LAMP1+ lysosomes.

4) lines 165-170, Fig. 3h. The authors state that "C9 aggregates form in a manner requiring…endosome acidification". As interference with endosome acidification using bafilomycin or NH4Cl reduced aggregation by only 30%, this statement should be amended. This statement was changed in the Results section.

5) lines 178,179, Fig. 4d,e,g: "…C9 became containing within LC3+ membranes…displaying markers specific for aggresomes." Two separate stainings, one for LC3-RFP, and one for HSP70, are shown. It is thus not clear whether LC3-RFP membranes also display HSP70. We have newly included images along with quantification of LC3B+ punctae colocalization with Hsp70 in Fig 4*h.*

6) lines 183,184: Fig. 4k: "AA197-270…colocalized with Thioflavin+Hsp70+aggresomes". This colocalization appears to be only partial. Please provide a quantification for this colocalization using a statistically significant number of cells. The reviewer is correct in noting partial AA197-270 colocalization with thioflavin, and quantification have been added to the new Figure 4*l*.

7) lines 248-265: In some cases, the results of specific experiments seem like a foregone conclusion. For example, the authors perform a proteomic analysis, looking for EB3 ubiquitin ligases binding to ZFYVE21, only to pick RNF34, a protein that they already identified as a ZFYVE21 binding protein in a previous publication (Li et al., Nat Commun, 2023). We thank the reviewer for this comment, and we provided improved clarification as to the results of the proteomic studies and the reasoning behind further study of RNF34:

We sought to identify E3 ubiquitin ligases utilized by ZFYVE21 to mediate C9 aggrephagy. We performed proteomic analyses of the ZFYVE21 interactome in ZFYVE21-GFP HUVECs treated with PRA for 2 hours. Three separate preparations yielded spectra for 3,347 proteins, 586 of which were uniquely upregulated by PRA (Supplementary Figure 2*g*). PRA-induced proteins included Rab5A/B/C, MAC proteins (C5/6/7/8α/8γ/9), and inflammasome proteins (NLRP3, casp1)[25] which previously appeared in prior MAC+Rab5+ proteomic datasets.[24] We additionally noted V-ATPase subunits (ATP6V1A/B2/C1/D/E1/H) and autophagy-related proteins (ATG3, ATG8G, P62), further supporting a role for vesicular acidification and ZFYVE21 within aggresomes, respectively.

We identified upregulated proteins in our proteomic datasets containing RING and HECT domains, domains known to show E3 ubiquitin ligase activity. We cross-referenced this list against proteins showing *z*-scores ≥2 in a prior genome-wide siRNA screen for NF-κB.[23] Among proteins identified, we focused on RNF34 which was chosen for analysis based on its strong spectral counts across multiple replicates and its concurrent detection in prior proteomic analyses of MAC+Rab5+ endosomes[24] and ForwardScatter^{Hi}ASC+Rab5+ vesicles.[26] RNF34 is an E3 ubiquitin ligase containing a FYVE domain allowing colocalization to Rab5+ endosomes and a RING domain enabling ubiquitinylation of immune-related substrates.[44,45] RNF34 has not been connected to macroautophagy but was previously found to directly bind ZFYVE21 in a process that enhanced RNF34 stability on Rab5+ endosomes in HUVECs.[26]

8) Fig. 6g: Please explain what is shown here in more detail. The legend is not really helpful. A new figure legend was made to better explain the updated Figure 6*g* which is Figure 6*i* in the revised manuscript. In this experiment, HUVECs transduced with mRFP-GFP-LC3 constructs and transfected with ZFYVE21 siRNA were analyzed by I.F. 72hrs after siRNA transfection following PRA treatment for the indicated times (*i*). GFP signal degrades with pH<6.0, and GFP+RFP+ signals indicate increased autophagosomes and not autophagolysosomes.

9) Fig. 9j: the labelling under the bar diagram is probably not correct. Please check. We apologize but cannot find Fig 9j in the Original Submission. Please clarify which figure, and we will make this correction.

10) Discussion: Given the wealth of data presented and their potential implications, the discussion appears to be rather sparse. The Discussion was truncated in the original submission to comply with word limit restrictions. We have placed the original Discussion intended for the paper in the revised manuscript.

Minor points:
1) abstract and text: "...promote NFkappaB…" please add "activity". This change was made throughout the manuscript.
2) lines 176,177: "In co-immunoprecipitations…C9 became ubiquitinylated". Please rephrase, as C9 is already ubiquitinylated before the co-IP. This change was made per the reviewer's request.
3) line 223: "became upregulated…". Here, probably "ZFYVE21" is missing. The reviewer is correct, and this change was made.
4) E.g. Fig. 3g: It is sometimes not clear what individual dots in diagrams represent, aggregates, cells or donors? Please add this information to the figure legends. The individual dots are now described in each figure legend.
5) Fig. 5j: The results showing CXCL7 and CXCL11 levels are not mentioned in the text.
Reviewer comments 1-5 listed above were addressed in the highlighted text in the manuscript. We believe these changes have improved the clarity of the manuscript.

Reviewer #3 (Remarks to the Author):

The manuscript by Song et al investigates the intracellular fate of complement membrane attack complex (MAC). In the manuscript the authors show that C9, a MAC protein, promotes loss of proteostasis to become an intracellular "alarmin". The authors show that C9 is internalised into Rab5 endosomes, promoting C9 aggregation and inducing NF-kB activation. This process seems to require ZFYVE21 , a Rab5 effector that links LC3 to RNF34-P62 complexes to mediate C9 aggrephagy. The authors confirm the involvement of ZFYVE21 by using gene deficient mice lacking ZFYVE21, that show reduced MAC-induced tissue injury in a skin model of chronic rejection. Overall, the manuscript is interesting, but the only novel element is the possible involvement of ZFYVE21 and its involvement is not very convincing. The senior author has previously demonstrated the internalisation of MAC via Rab5+ endosomes and the triggering of pro-inflammatory cytokines via the NF-kB pathway (PNAS 2015, 112(31):9689-9691, doi: 10.1073/pnas.1503535112). The reviewer is correct in mentioning our prior work showing that intracellular MACs elicited NF-κB, a finding that we mentioned in the Introduction that formed the basis for the present manuscript. The current manuscript expands on our prior findings to show that the MACPD/CDC domain of C9 forms aggregates in patient samples, *in vitro,* and *in vivo* to activate selective macroatuophagy, i.e., aggrephagy, to induce NF-kB. We further mechanistically show that aggrephagy proceeds via a ZFYVE21-RNF34-P62 signaling axis using novel ZFVE21 knockout mice. In this manuscript, we further define the immunostimulatory effects of MACs and EC-derived ZFYVE21. We believe that these are novel concepts which extend our findings in the above citation mentioned by R3.

The authors keep using Thioflavin as a stain for aggregated protein, but the specificity of thioflavin is also questionable. Thioflavin and Congo Red are histological stains that have traditionally been used to stain b-pleated sheets of amyloid. Their use in this manuscript to demonstrate aggregated protein raises questions regarding specificity – what exactly are the authors staining for when they see positive thioflavin staining? The reviewer is correct in that there is no stain that may specifically label C9 aggregates. We believe that C9 aggregates exist as outlined by the 6 points below:

1. We have directly visualized morphologic aggregates colocalizing with C9 in patient biopsies (Figure 1*a*), and in these samples we *a priori* excluded analyses of patients with primary or

secondary amyloidosis, and we avoided analyses of vascular sites known to contain confounding fibrillar structures including collagen fibrils in the adventitia and elastin fibrils in AV vessels.

2. To show the presence of C9 aggregates in human tissues, in new studies we fractionated sections of renal cell carcinoma tissues abundantly staining for C9 into soluble and insoluble fractions and noted that C9 appeared in the insoluble fraction. This aligned with *in vivo* studies using human artery xenografts subjected to IRI which similarly showed increased ratios of Insoluble:Soluble C9 (Figure 8*a*).
3. PRA-treated HUVECs similarly show morphologic aggregates containing C9 (Figure 2*a*), and we have reproducibly visualized colocalization of multiple dyes including thioflavin and Proteostat with C9 via I.F. in Figure 1g-l, 2e, 3d, 3i, 3k, 3l, 4i in the absence of collagen staining (Figure 2*e,f*).
4. We have observed C9+Thioflavin+ vesicles in Rab5, Rab7, and LAMP1 populations via vesicular flow cytometry.
5. Expression of the MCPF/CDC region of C9 also shows colocalization with thioflavin (Figure 4*l*).
6. C9 predominantly occurs within the insoluble fraction (Figure 3*g*, 3*e*, 3*f*) and acquires proteinase K resistance (Fig 2*k*).

While we cannot identify the precise nature of all proteins labelled by thioflavin, we believe based on the above that thioflavin staining labels C9 aggregates. We have also added the following text to the Discussion to convey the reviewer's point:

Thioflavin may stain fibrillar structures including amyloid proteins, collagen, and elastin. To obviate confounding staining, we excluded analyses of biopsies with either primary or secondary amyloidosis, and we focused on tissue regions lacking protein fibrils including collagen and elastin. Using immune EM we detected protein aggregates containing C9; and we detected increased ratios of insoluble C9:soluble C9 *in vitro*, *in vivo*, and in human tissues indicating that C9 accounted for a component of thioflavin staining despite the limitations of this stain.

In this study, they demonstrate the C9 internalisation via Rab5 and then claim that C9 polymerises intracellularly although I am not convinced by the data shown. C9 is shown to aggregate in cell free solution at lower pH but that does not demonstrate that this aggregation occurs intracellularly and we don't actually have the whole MAC (C9 already aggregated) internalised into Rab5+ endosomes. The reviewer raises an important point which is vital to our proposed hypothesis. To show that C9 aggregation occurs intracellularly, we performed 4 studies:

1. We blocked MAC internalization using 2 pharmacological agents, Pitstop and Dynasore. These treatments caused C9 to accumulate at the cell membrane and this had the effect of reducing thioflavin staining (Figure 3*d*).
2. In a corollary experiment, we assessed ratios of insoluble C9:soluble C9 after Pitstop2 treatment. We reproducibly observed that Pitstop2 caused increased insoluble C9:soluble C9 ratios, supporting the notion that C9 aggregates formed intracellularly (Figure 3*e*).
3. We blocked MAC internalization via a second method, via transfection of DNM2 siRNA. DNM2 siRNA phenocopied the above, increasing insoluble C9:soluble C9 ratios, further supporting that C(aggregates formed intracellularly (Figure 3*f*).
4. We stained PRA-treated HUVECs with Proteostat, a non-permeant dye. In the absence of permeabilization, we detected minimal Proteostat staining despite the presence of surface C9

staining on HUVECs. With the addition of a permeabilization step, we observed strongly increased C9+Proteostat+ staining, indicating that intracellular C9 became targets for Proteostat dye binding and that intracellular but not surface-derived C9 formed aggregates (Figure 3*g*).

These 4 studies supported the notion that C9 aggregates formed intracellularly. As below, we performed an additional 4 experiments to show that C9 entered into the endolysosomal system.

Having said that, I also feel that the quality of the images is poor and does not clearly show what the authors want to show. In order to properly visualise C9 aggregation in intracellular organelles high resolution fluorescent microscopy should be employed. To address this concern, we have inserted larger and higher resolution I.F. images for all figures in the manuscript, and we have separated select images into individual channels to enable better visualization of three-color colocalization which we have placed in the Supplementary Information section. We have applied a background subtraction step in all images to improve image quality, particularly in live cell imaging studies, and we have additionally inserted insets for select figures to further improve visualization of I.F. stains. We have provided quantifications for I.F. images shown inclusive of patient biopsies.

Also a time-lapse video of fluorescently labelled C9 would be very helpful in this case, so that the authors can demonstrate internalisation of C9 through Rab5, aggregation (which can be determined by the increase in fluorescent intensity) and then interaction with ZFYVE21??? Biochemical analysis showing C9 aggregation in Rab5+ organelles is also essential to convince this reviewer. The authors should isolate the organelle fraction using differential centrifugation and biochemically analyse the contents of the organelles at different time points (i.e. look for C9, Rab5, ZFYVE21, LC3, etc). The reviewer raises another valuable point, and to test C9 entry into the endolysosomal system:

1. We performed new live cell imaging studies. pHrodo is a pH-sensitive dye that fluoresces when pH<6.0. We treated HUVECs with PRA spiked with exogenous pHrodo-labeled C9, and this mixture was used to treat Rab5-GFP HUVECs. Within 5 minutes, we reproducibly visualized increased pHrodo+Rab5+ punctae. We wish to note that pHrodo-labeled C9 showed increased fluorescence in Rab5 endosomes whose has been described to be pH 4.5-5.5, thus showing entry of C9 into the acidified endolysosomal system (Figure 3*h,* Supplementary Movie 1).

2. In a second live cell experiment, we treated ZFYVE21-GFP HUVECs with AF647-labeled C9 and observed ZFYVE21+C9+ vesicle colocalization over time (Figure 6*e*, Supplementary Movie 2). As ZFYVE21 colocalizes to Rab5+ endosomes (PMCID: PMC2898296, PMCID: PMC6529429), these data further support C9 entry into the endolysosomal system.

3. To further visualize C9 aggregates within the endolysosomal system, we performed I.F. staining of native endolysosomes including Rab5, Rab7, and thioflavin. In these studies we visualized increased C9 colocalization with these endolysosomes, in particular, Rab7 (Figure 3*i*).

4. We newly performed FACS analyses of gated vesicular populations in PRA-treated HUVECs in Figure 3*j*. In these studies, we gated on C9+ vesicles and found that thioflavin staining increased in by 49%, 42%, and 51% within Rab5, Rab7, and LAMP1, vesicles, respectively, at 1 hour following PRA treatment. This demonstrated the presence of C9+Thioflavin+ signal within intracellular vesicles representative of the endolysosomal system whose intraluminal pH is known to be <6.

5. To further support the notion that C9 aggregates formed within the endolysosomal system, we transduced HUVECs with Rab5 WT or Rab5 DN constructs and observed that loss of activity for Rab5 whose effectors are required for downstream endosome maturation and progression the through the endolysosomal system almost completely ablated Thioflavin+C9+ staining (Figure 3*k*).

These 4 studies support the notion that C9 enters the endolysosomal system.

The only novel element of the manuscript is the possible involvement of ZFYVE21, and again I don't find the data presented particularly convincing. The authors do utilise mouse models to demonstrate this, although I think CRISPR knockout of several components of this pathway (ZFYVE21, LC3B etc) on HUVECs would have been much better. With regards to the former concerns as to lack of novelty, we have performed a comprehensive PubMed literature search. Based on this search, we believe there are numerous novel concepts raised in our manuscript that have not been previously reported. These include:

1. C9 aggregates forms in patient biopsies, in PRA-treated HUVECs, and anti-H2^b-treated mice.
2. The MACPF/CDC region of C9 is sufficient for aggregate formation.
3. C9 aggregates form in the endolysosomal system.
4. C9 stimulates aggrephagy to activate NF-κB.
5. ZFYVE21 is a direct LC3B binding partner and functions as an adaptor, linking LC3B on autophagosome membranes to RNF34-P62 complexes that ubiquitinylate and degrade C9.
6. RNF34 ubiquitinylates and degrades C9 to mediate C9 aggrephagy.
7. C9 aggrephagy and ZFYVE21-associated signaling responses for C9 aggrephagy occurs *in vivo.*
8.Novel generation of mice with global and EC-specific loss of ZFYVE21 show a role for ZFYVE21 in C9 aggregraphy *in vivo*

Based on the above, we believe the manuscript contains many novel points which have not been previously reported.

To address the latter concern, we performed CRISPR/Cas9 gene deletions of ATG16L (Supplementary Figure 1*h*) and ZFYVE21 (Supplementary Figure 2*b*).

1. In ATG16L knockout cells treated with PRA in pulse-chase studies, we found that genetic loss of ATG16L reduced autophagic flux while potentiating levels of C9. This had the effect of reducing NF-κB (pP65)
2. In ZFYVE21 knockout cells, we noted that loss of ZFYVE21 similarly potentiated C9 levels while reducing autophagic flux (Supplementary Figure 2*b*). To support a role for ZFYVE21 in autophagy *in vivo,* we have newly generated Ella-Cre ZFYVE21fl/fl x LC3B-GFP-RFP autophagy reporter mice. Complementing *in vitro* findings, we found that genetic loss of ZFYVE21 in ECs decreased C9 autophagosomes *in vivo* in Figure 9*g*.

They claim that ZFYVE21 deficient animals demonstrate a reduced MAC-induced tissue injury in a skin model. I fail to see how they can make that claim when the tissue injury in this skin model can not be just MAC-induced but must involved other immunological elements. We apologize for this apparent misunderstanding. We agree with the reviewer, and we do NOT believe that MACs are able to induce tissue injury in the absence of immune cells. We instead believe that MAC increases the immunogenicity of ECs to allow immunological elements to potentiate tissue injury. New experiments in Figure 10 show that MAC-treated ECs increase effector responses of numerous immune cell types in both the myeloid and lymphoid cell populations as described above in our rebuttal to R1. In skin transplant studies into

mice having both lymphoid+myeloid cells (C57/Bl6), myeloid cells only (SCID/bg), and neither lymphoid or myeloid cells (SCID/bg+LC) in Figure 10*a,b,* MAC-bound ECs alone showed only a minor contribution to tissue injury assessed by epidermal thickening and CD45+ immune cell infiltrates and required contributions from both lymphoid and myeloid lineages. We hope that the above and expanded clarifications in the Results section will help to address R2's concern.

An interesting paper, confirming some of the author's previous findings, but lacking the evidence for the novel part of the claims, regarding the involvement of ZFYVE21 and autophagy. As the reviewer notes, the findings in this manuscript are based entirely on our prior results, indicating reproducibility and robustness. We have applied myriad cell-free, *in vitro,* and *in vivo* approaches as well as patient specimens, and these findings are supplemented by unbiased screens (limited siRNA screening, RNA seq studies, proteomic studies) to support conclusions regarding the involvement of ZFYVE21 and autophagy. Our *in vitro* approaches supporting a role for ZFYVE21 in C9 aggrephagy include biochemical, RNA-based, protein-based, and functional studies and have incorporated technically challenging assays including CRISPR/Cas9 and live cell imaging using gain- and loss-of-function approaches. Our *in vivo* studies utilize 3 separate mouse models (Figure 9) and utilize 2 genetic ZFYVE21 knockout strains (Figure 10) and a newly generated LC3B-GFP-RFP autophagy reporter strain. Our myriad approaches both *in vitro* and *in vivo* were intended to generate complementary data to support our claims regarding ZFYVE21 and autophagy.

We believe that the new, extensive data we have generated and text revisions have strengthened the manuscript, and we are appreciative for the feedback from the Editor and Reviewers. Thank you for taking the time to review this manuscript.

Sincerely,

Dan Jane-wit, MD, PhD

04/20/2026

We provide the following revisions in response to reviewer comments. We apologize for the delay in returning the manuscript back for revision due to personnel changes.

Reviewers' comments:

Reviewer #1 (Remarks to the Author):

This manuscript is much improved and represents a tour de force investigation of non-cytolytic immunogenicity of MAC. The data are robust and span both in vitro and precise in vivo studies that clearly dictate the immune cells involved in non-cytolytic functions of MACs and the molecular players completing the steps to activate the C9 based aggrephagy leading to NF-KB signaling and EC activation.

The authors have more than adequately addressed my prior concerns around innate and adaptive immunity in the C9/MAC driven inflammation. The additional studies of clinical samples in Figure 1 are noted. Likewise, new mechanistic data all contribute to a complete and interesting story that opens up new avenues for therapeutics to address antibody mediated pathologies in transplantation and potentially other antibody based immunopathologies.

I commend the others on their study.

We thank the reviewer for these positive comments.

Reviewer #2 (Remarks to the Author):

The authors have addressed all of my raised points sufficiently. A small detail regarding my major point 9): "Fig. 9j: the labelling under the bar diagram is probably not correct. Please check." This included a typo, actually meaning Fig. 8j. Please check the labelling there.

We appreciate the reviewers feedback. The manuscript has been heavily modified, and we find former Fig 9j (current Fig 9i) to be labeled correctly. We have rechecked the text to ensure that the callout in the text for Fig 9i is correct. Thank you for your careful read of the paper.

Reviewer #3 (Remarks to the Author):

I feel that the re-submitted manuscript by Dan Jane-wit and colleagues is much improved from the original submission and the authors have made an effort to address all the reviewers' comments.

They have provided evidence that Membrane Attack Complexes (MACs) are internalized in Rab5+ vesicles to promote aggrephagy that results in NF-KB activation. These findings have been described in their past work, Fang et al. 2019. Nat. Comm. and Li et al. 2023. Nat. Comm. thus I have been struggling with the novelty of

the findings. The involvement of ZFYVE21 is a novel element and the authors have taken the comments on board to strengthen that finding with CRISPR knockouts.

We believe there may be an objective misunderstanding regarding findings in our prior published work. The citations provided by R3 regarding our published Nature Communications papers are incorrect.

The Nature Communications papers showed that ZFYVE21 mediates lipid (Fang et al, 2019) and protein remodeling (Li et al, 2023) of Rab5+ endosomes to activate inflammasomes. We have NOT focused on 'aggrephagy' in our prior studies in **any** context. In performing a literature search in PubMed, we are unable to identify manuscripts including our own published work describing C9 aggregates or C9 aggrephagy.

We wish to re-iterate that the core finding of the paper is NOT inflammasomes but rather that MAC component proteins acquire intrinsic immunogenicity by forming intracellular aggregates that activate aggrephagy. This mechanism is entirely separable from inflammasome activity shown previously in our prior work published in Nature Communications. To our knowledge, this concept has not been described by *any* other group including our own. We have extensively rewritten the Introduction section in light of R3's comments to clarify the above. This manuscript presents a completely novel finding for crosstalk between immune activation and cellular homeostasis pathways that have consequences for human disease mechanisms.

While we believe there may have been objective misunderstanding regarding the novelty of our present manuscript, we do not wish to minimize R3's concerns for novelty. In good faith we have performed new experiments listed below.

Figure 1j
Figure 2b
Figure 2h
Figure 2i
Figure 2j
Figure 2k
Figure 3b
Figure 3c
Figure 3d
Figure 3e
Figure 3f
Figure 3g
Figure 3h
Figure 3i
Figure 3j
Figure 3k
Figure 3l
Figure 4*d*
Figure 4*e*
Figure 5*a*

In our new studies, we made the 4 conceptual advances below, providing increased mechanistic insights into intracellular C9 aggregates:

1. To generalize findings of C9 aggregates in human tissues, we newly analyzed tissues from patients with renal cell carcinoma (RCC), a condition causing extensive MAC formation (Supplementary Fig 1*e*), to show insoluble C9 in RCC but not healthy control biopsies (Figure 1*j*).

2. We performed new electron microscopy studies to colocalize C9+ aggregates within large intracellular structures containing numerous vesicles and showing morphologic aggregates, C1q complement complexes, but not ribosomes (Figure 2*b,h-k,* Figure 5*a*).

3. We performed new proteomic studies and identified a novel C9 binding protein, NUMBL, promoting C9 internalization and C9 aggregate formation. We find that NUMBL is post-translationally stabilized on Rab35 endosomes where it functions as a Rab35 effector protein to bind C9 to mediate its internalization. We identify 2 binding sites on C9 that are required for NUMBL binding (Figure 3*b-g*).

4. We performed biochemical and cell-based studies to show that NUMBL is required for endolysosomal acidification to promote C9 aggregates (Figure 4*d,e*).

5. We found that NUMBL became rapidly recruited to Rab35+ endosomes (Figure 3*e,f*) which shielded and sequestered NUMBL from proteasome degradation (Figure 3*j*). Rab35 activity was required to

stabilize NUMBL (Figure 3*j,k*) and to induce C9 internalization (Figure 3*i*). Based on this, we tested Rab35-NUMBL interactions and found using cell-free (Figure 3*l*) and cell-based (Figure 3*j-k*) approaches that NUMBL is a novel Rab35 effector protein.

In addition, the authors have made an attempt to generate time-lapse video of fluorescently labelled C9 to demonstrate internalisation of C9 through Rab5. I do not find the actual videos convincing - but the stills in the figures seem to demonstrate the point. I think the authors need to quantify the co-localisation in those images in order to strengthen their point of C9 internalisation in Rab5 endosomes. Furthermore, as I have previously suggested it would be good to also show the interaction (co-localisation) with ZFYVE21 given that this one of the most novel findings of the work. These data were shown previously in the prior submission, but we wish to reiterate that the data in Figure 4*a* shows quantification of C9 within acidified Rab5+ endosomes and quantification of C9 aggregates within endolysosomes including Rab5, Rab7, and LAMP1 vesicles using I.F. and vesicular flow cytometry (Figure 4*b-c*).

Also, we wish to reiterate that we previously showed colocalization of C9+ aggregates with ZFYVE21 in aggresomes in Figure 6*c-d* and in live cell imaging in Figure 6*e*. As the manuscript has gotten quite large over the prior revisions with many figures, we understand the basis for why these data may have been missed.

Overall an improvement to the original submission and an honest attempt to address all of the reviewers' comments. Thank you for this positive comment. Our current manuscript newly demonstrates immunogenicity of intracellular C9 aggregates and demonstrates translational relevance of this novel concept in patients with multiple disease conditions (Figure 1) and in 3 *in vivo* models (Figure 8-10). Our findings are conceptually novel with **no** publications to our knowledge (including our own) describing immunogenic MAC aggregates promoting aggrephagy.

We would like to invite the reviewers, editors at Nature Communications, or an independent mediator to verify that existing published papers (including our own prior work) have not described C9 aggregates or C9 aggrephagy in **any** context.

We hope the reviewers and the editor can agree that the entirety of the work is novel, that these extensive revisions have alleviated prior concerns, and that this manuscript is suitable for publication at Nature Communications. As before, we remain very receptive and appreciative for your feedback. Thank you for considering this revised manuscript.

Sincerely,

Dan Jane-wit, MD, PhD

Associate Professor
Dept of Cardiology
Yale New Haven Hospital
Vascular Biology & Therapeutics Program
10 Amistad St, room 437C
New Haven, CT 06519
P: 216-571-4157

06/11/2026

We thank the reviewers for taking the time to review our manuscript and for their constructive comments. We believe that the reviewer comments have undoubtedly improved the quality of the manuscript during the revision process.

Reviewer #2 (Remarks to the Author):

I am very satisfied with the current version of the manuscript and recommend acceptance.

Thank you for this comment and for taking the time to review the manuscript.

Reviewer #3 (Remarks to the Author):

I feel that the re-submitted manuscript by Dan Jane-wit and colleagues is much improved from the original submission and the authors have made an effort to address all the reviewers' comments.
Although I had previously struggled to see the novelty of their findings, in the current version the authors have gone to great lengths to stress the novelty. The introduction is almost entirely re-written to present the new findings. They have identified NUMBL as a Rab35 effector that binds to C9 and promotes its internalisation. Once in endolysosomes, C9 aggregates are targeted for aggrephagy in a process that activates NF-kB. The importance of ZFYVE21 and RNF34 in this process is highly novel. The authors also use mice in order to detect these C9 aggregates in vivo. The new version of the manuscript clearly demonstrates the novelty of the findings demonstrating immunogenic MAC aggregates promoting aggrephagy I feel that the extensive revisions have alleviated prior concerns that I had regarding novelty, and that this manuscript is now suitable for publication to Nature Communications

We have made substantial changes to the manuscript in an effort to better convey its novel aspects. We are appreciative of the reviewer's comments.

Sincerely,

Dan Jane-wit, MD, PhD

Associate Professor
Dept of Cardiology
Yale New Haven Hospital
Vascular Biology & Therapeutics Program
10 Amistad St, room 437C
New Haven, CT 06519
P: 216-571-4157
